# The gene-rich genome of the scallop *Pecten maximus*

**DOI:** 10.1093/gigascience/giaa037

**Published:** 2020-04-30

**Authors:** Nathan J Kenny, Shane A McCarthy, Olga Dudchenko, Katherine James, Emma Betteridge, Craig Corton, Jale Dolucan, Dan Mead, Karen Oliver, Arina D Omer, Sarah Pelan, Yan Ryan, Ying Sims, Jason Skelton, Michelle Smith, James Torrance, David Weisz, Anil Wipat, Erez L Aiden, Kerstin Howe, Suzanne T Williams

**Affiliations:** 1 Natural History Museum, Department of Life Sciences,Cromwell Road, London SW7 5BD, UK; 3 University of Cambridge, Department of Genetics,Cambridge CB2 3EH, UK; 4 The Center for Genome Architecture, Department of Molecular and Human Genetics, Baylor College of Medicine, Houston, TX 77030, USA; 5 The Center for Theoretical Biological Physics, Rice University, 6100 Main St, Houston, TX 77005-1827, USA; 7 Wellcome Sanger Institute, Cambridge CB10 1SA, UK; 9 School of Computing, Newcastle University, Newcastle upon Tyne NE1 7RU, UK; 10 Institute of Infection and Global Health, Liverpool University, iC2, 146 Brownlow Hill, Liverpool L3 5RF, UK; 11 Shanghai Institute for Advanced Immunochemical Studies, Shanghai Tech University, Shanghai, China; 12 School of Agriculture and Environment, University of Western Australia, Perth, Australia

**Keywords:** scallop, bivalve, mollusc, genome, domoic, neurotoxin

## Abstract

**Background:**

The king scallop, *Pecten maximus*, is distributed in shallow waters along the Atlantic coast of Europe. It forms the basis of a valuable commercial fishery and plays a key role in coastal ecosystems and food webs. Like other filter feeding bivalves it can accumulate potent phytotoxins, to which it has evolved some immunity. The molecular origins of this immunity are of interest to evolutionary biologists, pharmaceutical companies, and fisheries management.

**Findings:**

Here we report the genome assembly of this species, conducted as part of the Wellcome Sanger 25 Genomes Project. This genome was assembled from PacBio reads and scaffolded with 10X Chromium and Hi-C data. Its 3,983 scaffolds have an N50 of 44.8 Mb (longest scaffold 60.1 Mb), with 92% of the assembly sequence contained in 19 scaffolds, corresponding to the 19 chromosomes found in this species. The total assembly spans 918.3 Mb and is the best-scaffolded marine bivalve genome published to date, exhibiting 95.5% recovery of the metazoan BUSCO set. Gene annotation resulted in 67,741 gene models. Analysis of gene content revealed large numbers of gene duplicates, as previously seen in bivalves, with little gene loss, in comparison with the sequenced genomes of other marine bivalve species.

**Conclusions:**

The genome assembly of *P. maximus* and its annotated gene set provide a high-quality platform for studies on such disparate topics as shell biomineralization, pigmentation, vision, and resistance to algal toxins. As a result of our findings we highlight the sodium channel gene *Nav1*, known to confer resistance to saxitoxin and tetrodotoxin, as a candidate for further studies investigating immunity to domoic acid.

## Context

**Figure 1:**
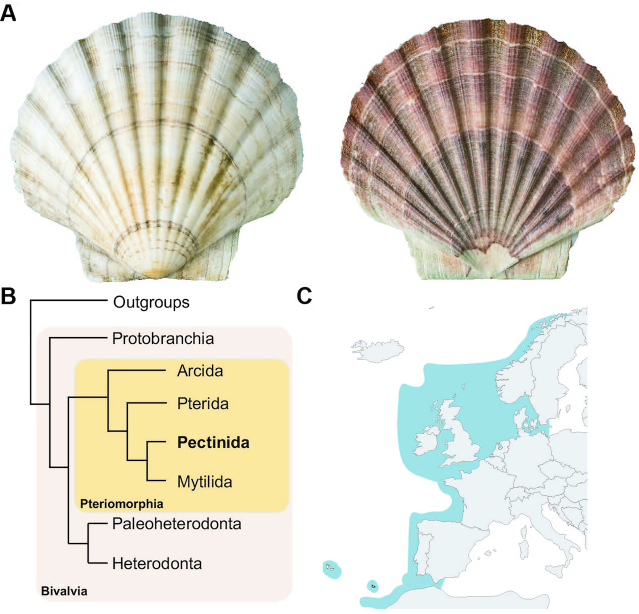
A, Photo of both valves of the shell of *Pecten maximus*, from the specimen sequenced in this work (NHMUK 20170376). B, Diagrammatic cladogram illustrating the phylogeny of the Bivalvia (after Gonzalez et al. [37]), showing the major sub-classes of Bivalvia and (boxed in yellow) the major divisions of the Pteriomorphia. *Pecten maximus* is a member of the superfamily Pectinoidea, which includes Pectinidae (scallops), Propeamussiidae (glass scallops), and Spondylidae (spiny oysters), and together with their close relatives (Anomioidea, jingle shells; Dimyoidea, dimyarian oysters; and Plicatuloidea, kittenpaw clams) these superfamilies form the order Pectinida. C, Distribution map of *P. maximus*, showing range (dark blue) of species across northern Europe and surroundings (map from simplemaps, distribution according to [2]).

Scallops are bivalve molluscs (Pteriomorphia, Pectinida, Pectinoidea, Pectinidae; Fig. 1A and B), found globally in shallow marine waters, where their filter-feeding lifestyle helps perform a variety of ecological functions [1]. There are ∼400 living scallop species [2], and of these, *Pecten maximus* (Fig. 1A), also known as the king scallop, great scallop, and St James scallop, is perhaps the best-studied European species. *Pecten maximus* is found around the coast of western Europe from northern Norway to the Iberian Peninsula (Fig. 1C) where it is locally common in many areas, and it can occasionally be found more distantly in West Africa and on mid-North Atlantic islands [2]. It is commercially fished across its range, most heavily around France and the United Kingdom [3, 4], and is the most valuable single-species fishery in the English Channel with ∼35,000 tonnes of international landings reported in 2016 [4]. It has also been cultivated in aquaculture, particularly in the United Kingdom, Spain, Norway, and France, although with limited commercial production [5, 6]. It is an important part of the ecosystems within which it occurs, performing key roles in food webs, both as a prey species and more indirectly by cycling nutrients during filter feeding [1].

Previous studies in this species have aimed to elucidate its population dynamics, swimming behaviour, visual systems, and reproduction (e.g., [7–10]). Of particular interest to medicine, fisheries management, and molecular biology is the means by which this species is resistant to neurotoxins such as saxitoxin (STX) and domoic acid (DA). DA and STX are potent neurotoxins produced by certain species of phytoplankton, including dinoflagellates and diatoms, which may be present in large blooms [3]. Some shellfish (e.g., scallops, *P. maximus;* mussels, *Mytilus edulis*; cockles, *Cerastoderma edule*; razor clams, *Siliqua patula*), fish (e.g., anchovy, *Engraulis mordax*; European sardine, *Sardina pilchardus*; and Pacific halibut, *Hippoglossus stenolepis*), and crabs (e.g., *Cancer magister*) accumulate algal neurotoxins by filtration of phytoplankton or by ingestion of contaminated organisms, with species-specific accumulation rates [11–13]. In humans, ingestion of DA or STX has been associated with gastrointestinal and neurological symptoms [14, 15]. In severe cases, poisoning by DA may lead to death or permanent memory loss, a syndrome known as amnesic shellfish poisoning (ASP), and in the case of STX, paralysis (paralytic shellfish poisoning [PSP]) [16]. Curiously, however, shellfish and fish that routinely accumulate algal toxins are often able to do so without apparent effect on their health [17, 18]. The resistance of *P. maximus* in particular, and of bivalve molluscs more generally, to these potent toxins is of keen interest to fisheries groups, health care providers, and molecular biologists, yet the genetic mechanism behind this remains unknown. Detailed investigation into this phenomenon, along with many others, would be greatly aided by a genome resource.

At the time of writing, 9 bivalve genomes are available, with those of the Pacific oyster *Crassostrea gigas* [19] and the pearl oyster *Pinctada fucata* [20] in particular having been used for a variety of investigations into bivalve biology. Scallops have been the subject of genome sequencing projects in the past, with genomes published for 3 species, *Azumapecten farreri* (as *Chlamys*) [21] and *Mizuhopecten yessoensis* (as *Patinopecten*) [22] from the subfamily Pedinae, and *Argopecten purpuratus* from the subfamily Pectininae [23]. Other sequenced genomes for pteriomorph bivalves include those of the Sydney rock oyster *Saccostrea glomerata* [24], eastern oyster *Crassostrea virginica* (unpublished, but see [25]), and the mussels *Mytilus galloprovincialis* [26], *Limnoperna fortunei* [27], *Gigantidas platifrons* (as *Bathymodiolus*), and *Modiolus philippinarum* [28]. There are also extant resources for more distantly related bivalves including the razor clam *Sinonovacula constricta* [29], snout otter clam *Lutraria rhynchaena* [30], blood clam *Anadara broughtonii* (as *Scapharca*) [31], Manila clam *Ruditapes philippinarum* [32], and the freshwater mussels *Venustaconcha ellipsiformis* [33], *Dreissena rostriformis* [34], and *Dreissena polymorpha* (McCartney et al. [35]). Of these resources, only the assemblies for *S. constricta, C. virginica*, and *S. broughtonii* are of chromosomal quality, and the scaffold N50 of the other resources varies widely.

These studies demonstrate that bivalve genomes are often 1 Gb or more in size, and generally exhibit large amounts of heterozygosity, related to their tendency to be broadcast spawners with excellent dispersal capabilities, resulting in large degrees of panmixia. Gene expansion has been noted as a characteristic of the clade, with some species exhibiting tandem duplications and gene family expansions, particularly in genes associated with shell formation and physiology (e.g., HSP70 [36]).

Here we describe the genome of the king scallop, *P. maximus*, which has been assembled from Pacific Biosciences (PacBio), 10X Genomics, and Hi-C libraries. It is a well-assembled and complete resource and possesses a particularly large gene set, with duplicated genes making up a substantial part of this complement. This genome and gene set will be useful for a range of investigations in evolutionary genomics, aquaculture, population genetics, and the evolution of novelties such as eyes and colouration, for many years to come.

## Methods

### Sample information, DNA extraction, library construction, sequencing, and quality control

A single adult *Pecten maximus* (NCBI:txid6579; marinespecies.org:taxname:140712) was purchased commercially, marketed as having been collected in Scotland. The shell was preserved and is deposited in the Natural History Museum, London, with registration number NHMUK 20170376. The adductor muscle was used for high molecular weight DNA extraction using a modified agarose plug–based extraction protocol (Bionano Prep Animal Tissue DNA Isolation Soft Tissue Protocol, Bionano Genomics, San Diego, CA, USA). DNA was cleaned using a standard phenol/chloroform protocol (phenol: chloroform: isoamyl alcohol 25:24:1, followed by centrifugation and ethanol precipitation), concentration determined with a Qubit high sensitivity kit, and high molecular weight content confirmed by running on a Femto Pulse (Agilent, Santa Clara, CA, USA).

**Figure 2:**
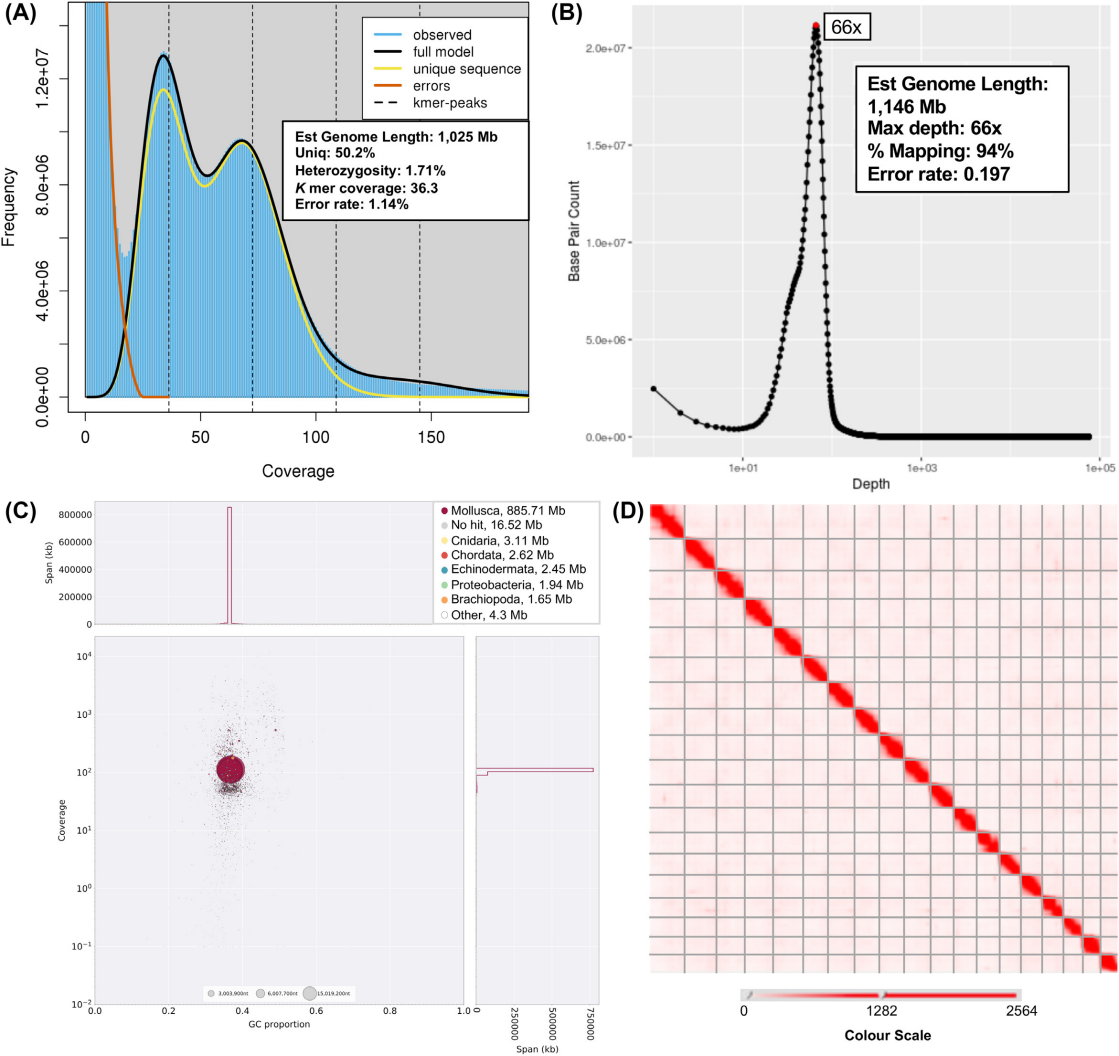
A, Genomescope2 [49] plot of the 21-mer *k-*mer content within the *Pecten maximus* genome. Models fitted and resulting estimates of genome size and read data as shown on figure. B, Base pair count by depth in PacBio data, determined using PBreads/Minimap2. C, Blobplot [50] of content of the *P. maximus* genome. Note that little-to-no contamination of the assembly can be observed, with the small amount of sequence annotated as non-metazoan mirroring the metazoan content in GC content and average coverage. Additional Blobplot plots and data, including those separated by phylum/superkingdom, can be found in Supplementary File 2. D, Hi-C contact map based on assembly created using 3D-DNA and Juicebox Assembly Tools (see [51] for an interactive version of this panel).

PacBio and 10X Genomics linked-read libraries were made at the Wellcome Sanger Institute High-Throughput DNA Sequencing Centre by the Sanger Institute R&D and pipeline teams using established protocols. PacBio libraries were made using the SMRTbell Template Prep Kit 1.0 and 10X libraries using the Chromium Genome Reagent Kit (v2 Chemistry). These libraries were then sequenced on Sequel 1 and Illumina HiSeq X Ten platforms, respectively, at the Wellcome Sanger Institute High-Throughput DNA Sequencing Centre. The raw data are available from the European Nucleotide Archive, with accession number ERS3230380. Hi-C reads were created by the DNA Zoo Consortium ( www.dnazoo.org) and submitted to NCBI with accession number SRX6848914. Read quality, adapter trimming, and read length were assayed using NanoPlot and NanoComp (PacBio reads) [38] and FastQC (10X reads, FastQC, RRID:SCR_014583) [39] (Supplementary File 1 [ 40, 41]). PacBio libraries provided ∼65.9× coverage of this genome; 10X reads and Hi-C provided a further 113.7× and 63.4× estimated coverage, respectively, assuming a genome size of 1.15 Gb as estimated from our reads (see Fig.   2). A summary of statistics relating to these reads can be found in Table 1.

**Table 1:** Libraries sequenced and used in assembly, with accession numbers

Library type	No. of sequencing runs	No. of reads	No. of bases (Gb)	GC %	Nominal coverage (1.15 Gb genome)	Accessions
10X	4	433,117,392	130.8	39.5	113.7×	ERR3316025–ERR3316028
PacBio	13	7,246,290	75.8	39.0	65.9×	ERR3130278–ERR3130281, ERR3130284–ERR3130292
Hi-C	1	241,297,364	72.9	38.7	63.4×	SRX6848914

### Genome assembly

PacBio reads were first assembled with wtdbg2 v2.2 using the "-xsq" preset option for PacBio Sequel data [42]. The PacBio reads were then used to polish the contigs using Arrow (genomicconsensus package, PacBio tools). This was followed by a round of Illumina polishing using the 10X data, which consisted of aligning the 10X data to the contigs with longranger align, calling variants with freebayes (freebayes, RRID:SCR_010761) 1.3.1 [43] and applying homozygous non-reference edits to the assembly using bcftools-consensus [44]. Medium-range scaffolding was performed using Scaff10X v.4.2 [45]. Longer-range Hi-C–based scaffolding was then performed on the 10X assembly by the DNA Zoo Consortium using 3D-DNA [46], followed by manual curation of difficult regions by means of Juicebox Assembly Tools [47]. A further round of polishing with Arrow was performed on the resulting scaffolds, with reads spanning gaps contributing to filling in assembly gaps. This was followed by a further 2 rounds of freebayes (freebayes, RRID:SCR_010761) Illumina polishing. Finally, the assembly was analysed and manually curated by inspection using the gEVAL browser [48].

Full statistics regarding our assembly can be seen in Table 2. The assembly contains a total of 918,306,378 bp, across 3,983 scaffolds. The N50 is 44,824,366 bp, with 50% of the genome found in 10 scaffolds. The Hi-C analysis identified that *P. maximus* possesses 19 pairs of chromosomes, in agreement with a prior study [52], and these are well recovered in our assembly, with 844,299,368 bp (92%) of our assembly in the 19 biggest scaffolds, the smallest of which is 32,483,354 bp, and the largest 60,076,705 bp in length; only 0.08% of the assembly is represented as Ns (691,874 bp). The assembly was screened for trailing Ns, and for contamination against databases of common contamination sources, adapter sequences, and organelle genomes derived from NCBI (using megaBLAST algorithm, requiring *e*-value ≤1e−4, sequence identity ≥90%, and for organelle genome comparisons, match length ≥500 [53]). This process identified no contamination. The Hi-C contact map for the final assembly (Fig. 2D) demonstrates the integrity of the chromosomal units. The interactive version of the contact map is available at [51] (powered by Juicebox.js [54]) and on the DNAzoo website [55]. Our assembly is the most contiguous of all published bivalve genome assemblies to date (Table 3).

**Table 2:** Basic metrics relating to assembled genome

Total assembly length (bp)	918,306,378
GC content of scaffolds	36.62%
Maximum scaffold length (bp)	60,076,705
N50 scaffold length (bp)	44,824,366
N90 scaffold length (bp)	32,483,354
No. of scaffolds	3,983
No. of scaffolds in N50	10
No. of chromosomes	19
% genome, chromosome-length scaffolds	92%
N content, total (bp)	691,874

**Table 3:** Genomic assemblies of a number of marine bivalves, and summary statistics relating to these assemblies

Family	Species	GC content (%)	Assembled length (Mb)	No. of scaffolds	Longest scaffold (Mb)	Scaffold N50 (Mb)	No. of missing BUSCOs (%)	Source
Pectinidae	*Pectenmaximus*	37	918.3	3,983	60.1	44.8	44 (4.5)	This work
Pectinidae	*Azumapecten farreri*	35	779.9	96,024	6.5	0.6	53 (5.5)	[21]
Pectinidae	*Argopecten purpuratus*	35	724.8	89,727	11.1	1.0	36 (4.2)	[23]
Pectinidae	*Mizuhopecten yessoensis*	34	987.6	82,659	7.5	0.8	53 (5.5)	[22]
Mytilidae	*Gigantidas platifrons*	30	1,658.2	65,662	2.8	0.3	38 (3.9)	[28]
Mytilidae	*Modiolus philippinarum*	32	2,629.6	74,573	0.7	0.1	55 (5.6)	[28]
Pteriidae	*Pinctada fucata*	33	815.3	29,306	1.3	0.2	45 (4.6)	[20, 36]
Ostreidae	*Crassostrea gigas*	30	557.7	7,659	2.0	0.4	38 (3.9)	[19]
Ostreidae	*Saccostrea glomerata*	33	788.1	10,107	7.1	0.8	56 (6.7)	[24]

These data, with comparison to Gastropoda, can be seen in Table 1 of Sun et al. [72].

### Assembly assessment

The total size of our assembly, 918 Mb, falls short of previous estimates of the genome size of *P. maximus*, with flow cytometry estimating a genomic C-value of 1.42 [56]. Assessments of genome size based on *k-*mer counting using Genomescope (10,000 cov cut-off) [57] suggest that the complete genome size is ∼1.025 Gb (Fig. 2A). Estimates using PacBio reads and Minimap2 [58], showing base pair count at each depth, put the genome size at 1,146 Mb, which is more in line with flow cytometry results. This discrepancy is likely to be caused by heterochromatic regions inaccessible to current sequencing technologies.

The expected genome size of *P. maximus* is slightly larger than many other sequenced bivalve species, and our assembly size (in base pairs) is in line with that of other sequenced scallop species (Table 3). It is, however, half the size of the genomes of the sequenced mussels *G. platifrons* and *M. philippinarum*. Scallops therefore have intermediate genome sizes on average when compared to other molluscs - larger than oysters such as *C. gigas* and gastropods such as *Lottia gigantea*, but smaller than mussels and cephalopods. The reasons for these differences in genome size are at present unclear but may include gene duplications, repetitive element expansions, and, in some cases, whole-genome duplications (WGDs) [59].

To confirm the efficacy of the contamination screen performed during the assembly process, we verified the absence of parasitic or pathogenic sources by creating a Blobplot (Fig. 2C) using Blobtools (Blobtools, RRID:SCR_017618) [50]. We observed very few scaffolds (1.94 Mb, or ∼0.21% of our assembly) with blast similarity to Proteobacteria, but with coverage values and GC content exactly mirroring the rest of the assembly. In the majority of these cases, the assignment to Proteobacteria will be due to a chance blast match with high similarity over a small region of the contig length, rather than actual bacterial origin (Supplementary File 2 [40, 41]). The vast majority of the assembly (885.71 Mb) was assigned to the clade Mollusca, as expected (Fig. 2C).

To assay assembly quality and completeness, we mapped our raw reads to the genome. Of the 10X Genomics paired-end reads, 94% (8.14 ×10^8^ of 8.66 × 10^8^ reads) mapped concordantly. Of our PacBio reads, 94% (71.13 × 10^9^ of 75.7 × 10^9^ bases) also mapped (Fig. 2B), indicating a well-assembled dataset, and one with little missing data.

The reasonably high level of observed heterozygosity calculated by GenomeScope (GenomeScope, RRID:SCR_017014) [ 57] from raw reads (1.71%, Fig.   2A) in the *P. maximus* assembly is a common phenomenon in broadcast-spawning marine invertebrates [60]. It should be noted that we used freebayes-polish on our final assembly when using this resource for studies focusing on genetic diversity, and no detectable heterozygosity will remain. In our raw reads, levels of heterozygosity in *P. maximus* were higher than those found in the Sydney rock oyster *Saccostrea* (0.51%), or the Pacific oyster *C. gigas* (0.73%). Both of these oyster samples were derived from selective breeding programmes, which would reduce heterozygosity compared to wild populations [24].

Repetitive elements have been noted as playing an important role in genome evolution in molluscs, and in bivalves in particular (e.g., [61]). We used RepeatModeler (RepeatModeler, RRID:SCR_015027) and RepeatMasker (RepeatMasker, RRID:SCR_012954) [62] to identify and mask regions of the genome containing previously identified or novel repetitive sequences (Table 4). With the caveat that not all repetitive elements have been classified, it seems that long terminal repeats (LTRs) are less common in *P. maximus* compared to other species (0.52%, cf. 1.35% in *S. glomerata* and 2.5% in *C. gigas*) but that short interspersed nuclear elements (SINEs) are more common (2.19%, cf. 0.09% in *S. glomerata* and 0.6% in *C. gigas*). A total of 27.0% of the genome was classified as repetitive elements, with 16.7% of the genome made up of elements not present in preconfigured RepeatMasker libraries (but likely shared with other bivalve species). While the genome of *P. maximus* is large by scallop standards, its size is not due to large amounts of repetitive elements because 27.0% is low compared to many other genome resources. For example, *C. gigas* has a repeat content of 36% [19], and *S. glomerata*, 45.0% [24].

**Table 4:** Repeat content of the *P. maximus* genome based on RepeatModeler and RepeatMasker analysis

Element	Count	Length occupied (bp)	% of genome
SINEs	125,121	20,067,275	2.19
MIRs	21,406	3,059,644	0.33
LINEs	86,373	26,983,591	2.94
LINE1	803	463,519	0.05
LINE2	4,883	2,601,659	0.28
L3/CR1	4,374	1,588,697	0.17
LTR elements	9,334	4,731,793	0.52
DNA elements	121,409	31,845,557	3.47
hAT-Charlie	1,312	394,533	0.04
TcMar-Tigger	4,548	1,478,364	0.16
Unclassified	612,341	153,700,734	16.74
Total interspersed repeats		237,328,950	25.84
Small RNA	4,096	563,615	0.06
Simple repeats	174,931	9,099,659	0.99
Low complexity	25,658	1,411,700	0.15
Total length (of 918.3 Mb):		247,513,725	26.95

### Gene prediction and annotation

Gene sequences were predicted using Augustus (Augustus: Gene Prediction, RRID:SCR_008417) annotation software [63], with 1 novel [40, 41] and several previously published *P. maximus* RNA sequencing (RNAseq) datasets [64, 65] used for training. The novel dataset was derived from 2 samples of *P. maximus* mantle tissue from the same specimen used for genomic DNA extraction. These were sequenced on an Illumina HiSeq to a depth of 338,910,597 reads. After initial trimming of poor-quality sequence and residual adapters with TrimGalore v0.6 [66], this library was assembled using Trinity (Trinity, RRID:SCR_013048) v2.8.4 [67] with all default settings. Following assembly, chimeric, fragmented, or locally misassembled transcripts were filtered using Transrate v1.0.3 [68], where "good" transcripts were retained, followed by DETONATE (DETONATE, RRID:SCR_017035) v1.11 with the bowtie2 option [69], where transcripts scoring <0 were discarded. Transcripts were then clustered using cd-hit-est v4.8.1 [70] at an identity threshold of 95% (-c 0.95 -n 8 -g 1), and the representative sequence of each cluster was retained. The non-masked genome was used as the basis for gene prediction, to avoid artefacts, missed exons, or missing gene portions caused by gene overlap with masked areas of the genome. Training was first performed using the aforementioned RNAseq datasets, as part of the AUGUSTUS pipeline (which incorporates BLAT alignment [71]). After training, the resulting hints file was submitted once more to Augustus for prediction, with options regarding untranslated regions (UTRs) and gene prediction on both strands set to “true.” The same messenger RNA files used for initial training were also provided to AUGUSTUS for this prediction step. Note that UTR prediction with AUGUSTUS is imperfect in non-model organisms, and UTR regions provided here are current best estimates and would benefit from full-length RNA sequencing (e.g., Isoseq, on the PacBio platform).

This annotation resulted in an initial set of 215,598 putative genes (with 32,824 genes having ≥2 alternative isoforms), resulting in 249,081 discrete transcript models. We filtered the initial gene set by comparing our gene models to 7 previously published bivalve resources (*A. purpuratus, A. farreri, M. yessoensis, C. gigas, P. fucata, G. platifrons*, and *M. philippinarum*) using Orthofinder2 (OrthoFinder, RRID:SCR_017118), and retained genes with orthologues shared with other species (57,574 genes, further details below). To ensure that we did not discard transcribed genes absent from other bivalves but present in our resource, we also retained those genes with an empirically determined “good” hit in the *nr* database, lenient enough to recover genes from more distantly related species but stringent enough to avoid chance similarity (23,541 genes, diamond blastp, –more-sensitive –max-target-seqs 1 –outfmt 6 qseqid sallseqid stitle pident evalue –evalue 1e-9 [73]), a total of 81,115 genes. However, we then removed from this combined total any genes that had a match within our identified repetitive elements (13,374 genes, tblastn, -evalue 1e-29 -max_target_seqs 1 -outfmt ‘6 qseqid staxids evalue’ [53]). This evalue cutoff was chosen after initial trials to include genes that mapped to *pol, env, tc3 transposase, Gag-Pol*, and *reverse transcriptase* genes in automated blast. This resulted in a final, 67,741-gene, curated set, of which 16,693 genes possess ≥1 alternative transcript. Full, curated and annotated gene sets in a variety of formats can be found in online repositories [40, 41].

This number, while still high in comparison to the number of genes found in many metazoan species, is comparable to the number of unigenes (72,187) in the *Argopecten irradians* resource [74]. To confirm the veracity of these gene models as transcribed genes, we mapped samples from a number of previously sequenced, independent RNAseq experiments to our gene models using STAR 2.7 [75] and the –quantMode GeneCounts option. This records only the reads corresponding to 1 gene, with no multimappers recorded, and is thus a highly stringent test of transcription. Of our 67,741 curated “high-confidence” gene models, 47,159 (69.6%) were transcribed in the novel mantle-specific RNA dataset presented in this article. From independent samples, 33,553 genes were transcribed in the mantle of the sole control sample from a previous heat stress experiment [64]. A total of 48,882 genes were expressed in 2 replicate late veliger controls from an experiment where embryos were exposed to a range of water conditions (varying pH, PRJNA298284) and 39,640 were expressed in MiSeq reads sampled from mixed adductor muscle, hepatopancreas, and male and female gonad tissue (PRJEB17629). In total, 57,368 of our 67,741 curated high-confidence gene models (84.7%) are supported by these independent RNAseq experiments, 54,153 (79.9%) of which were found in samples other than our novel transcriptome. These mapping results have been made available for download as Supplementary File 3 (40, 41. It should be noted that this is likely an underestimate of transcription, given that multi-mapping reads were discounted from consideration. If additional tissues and life stages were targeted, given the fact that these genes have known orthologues in closely related species (see Orthofinder2 results above), it is likely that almost all of our gene models would be found to be expressed.

The 84,866 transcripts in our high-confidence gene set (some genes possess >1 transcript) have a mean of 5 exons. This is fewer than that seen in *M. yessoensis* (7 exons on average) or *P. fucata* (6 on average) (Table S8, [22]). This may indicate a degree of fragmentation in our gene models (although that is not observed empirically), or alternatively, that some of the genes in our gene models have been copied via retrotransposition and lack introns, which would lower the average exon number and contribute to the high number of genes seen in this species.

We assayed the completeness of our gene set using BUSCO v2 (BUSCO, RRID:SCR_015008) [76], using metazoan gene sets. Of the 978-gene Metazoa dataset, 924 (94.5%) complete BUSCOs (of which 32 [3.3%] were duplicated), 10 incomplete (1.0%) BUSCOs, and 44 (4.5%) missing BUSCOs were recorded in genome mode, equating to a recovery of 95.5% of the entire BUSCO set. This is comparable to previously published bivalve resources (Table 3).

We have performed annotation of gene complements using 2 automated methods. BLAST annotation was performed with peptide sequences using DIAMOND against the *nr* database (locally updated 11 November 2019) with more lenient settings than used for curation of our gene models (tblastn, –more-sensitive –max-target-seqs 1 –outfmt 6 qseqid sallseqid stitle pident evalue –evalue 1e-3 –threads 4 [73]), with 88,824 of our unfiltered gene models recovering a hit, although this figure includes hits to repetitive elements removed in our curated dataset (Supplementary File 4, 40, 41). Of the 67,741 high-confidence genes, 59,772 possess a hit in the nr database (88.2%), indicating a highly annotatable dataset. We also used the KEGG-KAAS automatic annotation server, using peptide sequence and the Bidirectional Best Hit (BBH) method. The standard eukaryotic species set, complemented with *L. gigantea, Pomacea canaliculata, C. gigas, M. yessoensis*, and *Octopus bimaculoides* was used for annotation, with 14,495 of our gene models mapping to KEGG pathways (Supplementary File 5, 40, 41).

### Gene complement and expansion

**Figure 3:**
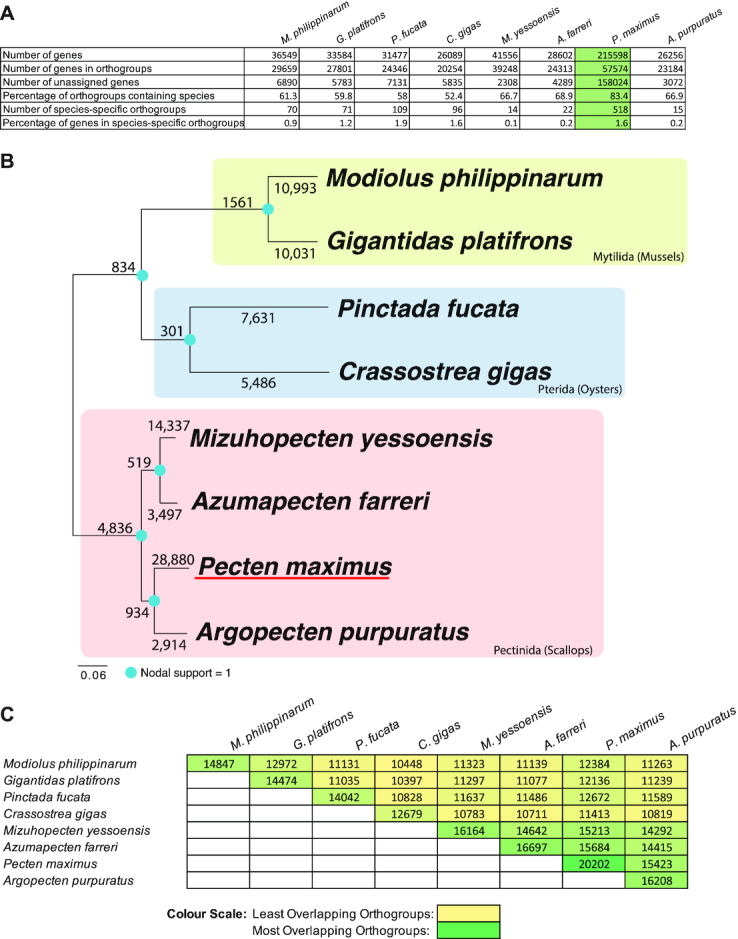
A, Orthofinder 2 [82] ortholog analysis of 8 sequenced marine bivalve species. *Pecten maximus* results shown in green. B, Phylogeny of bivalves using available marine bivalve genomes (generated from ortholog groups by STAG and displayed in Figtree), with root placed at midpoint. Blue dots indicate nodal support (=1 at every node). Numbers on internal nodes represent ancestrally shared duplications at the point of diversification. Numbers on leaf nodes indicate duplication events occurring solely in that taxon. C, Matrix showing numbers of overlapping orthogroups shared by the species examined. A colour scale has been applied to aid in identifying the most- and least-overlapping data sources.

We investigated the gene complement of *P. maximus* to understand the nature of the events that resulted in it and other scallops possessing a large number of annotated genes compared with related mollusc species. This analysis was performed predominantly using Orthofinder2 (-t 8 -a 8 -M msa -T fasttree settings and using only the longest transcript per gene for *P. maximus*, Fig. 3A). These statistics reveal that *P. maximus* exhibits little gene loss compared with other related species. The percentage of orthogroups containing *P. maximus* genes is very high (83.4%) compared to every other species examined. *Pecten maximus* has therefore lost fewer genes from the ancestrally shared cassette than any of the other species listed. *Pecten maximus* also possesses 518 species-specific orthogroups—comparatively more than any other species listed. These genes could be true novelties because they are not found in any of the 8 other species of bivalve examined here, but they may be derived from repetitive content, as the unfiltered *P. maximus* gene set was used as the basis of this comparison.

Using these results, we are also able to understand the prevalence of gene duplication across the phylogeny of bivalves. Gene duplication events were inferred from the orthogroup analysis and mapped onto the phylogeny of the 8 bivalve species examined here (Fig. 3B). We conclude that gene duplication events are common in extant species of bivalve, and some gene duplicates are shared by leaf nodes as a result of events in the stem lineage. However, duplications in *P. maximus* are particularly prevalent. With 28,880 unique duplications, *P. maximus* has more than double the number of duplicates of any other species, with *M. yessoensis* the next closest example. However, it should be noted that not all gene annotations were performed in an identical fashion, and particularly if genes have been missed in other species, e.g., through sparse RNAseq for gene prediction, this will negatively influence their counts in these results.

Of the genes that are shared with other lineages, *P. maximus* has a highly complete complement (Fig. 3C). No other species examined here possesses as many shared orthogroups in total or shares as many with other species. In pairwise comparisons, only the mussels *M. philippinarum* and *G. platifrons* show similar numbers of shared orthogroups with each other, but not with other species. This is consistent with the previous finding that the scallop *M. yessoensis* is closer in gene complement to the oysters *C. gigas* and *P. fucata* than the oysters are to one another [22], a fact reflected in early divergence of these 2 distantly related oyster species [77]. Scallops in general therefore have a better-conserved gene cassette compared to the ancestral genotype than exhibited in oysters.

We conclude that *P. maximus* has a well-conserved gene set, which has been added to substantially by gene duplication. Its large gene complement is therefore explained by a strong pattern of gene gain, coupled to very little gene loss.

### Hox genes

The prevalence of gene duplication within *P. maximus* led us to consider whether a WGD event had occurred in this lineage. As a test for this, we used the well-conserved Hox and Parahox gene clusters, which are normally preserved as intact complexes and duplicated in the presence of additional WGD events (e.g., [78, 79]).

**Figure 4:**
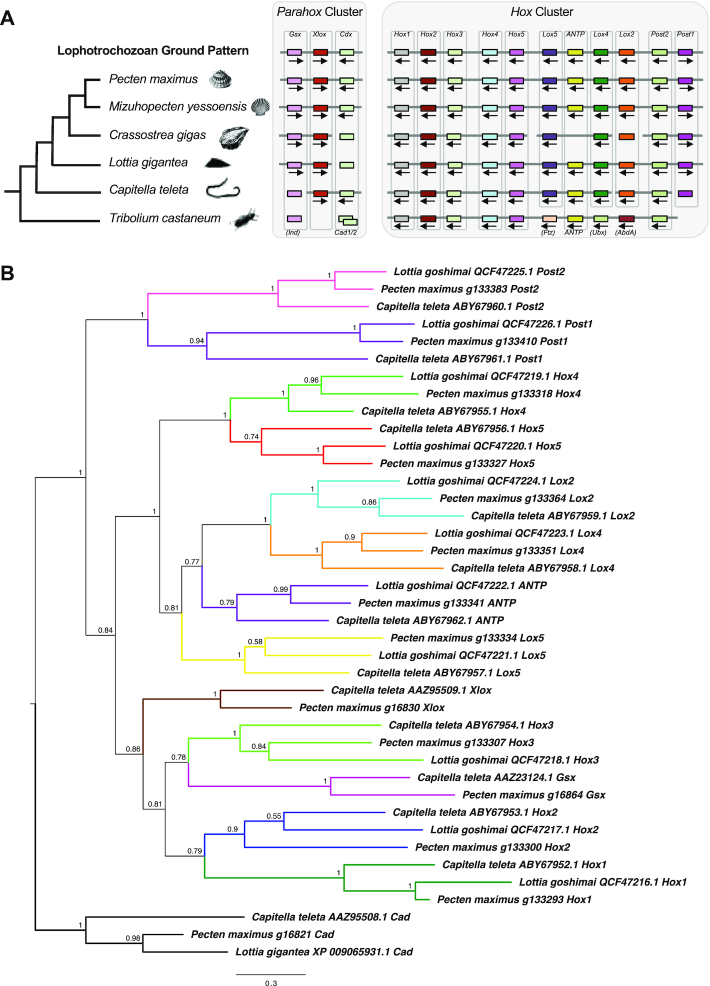
A, Diagrammatic representation of Hox and Parahox cluster chromosomal organization showing a shared pattern among selected Lophotrochozoan taxa (scallops *Pecten maximus* and *Mizuhopecten yessoensis*, Pacific oyster *Crassostrea gigas*, owl limpet *Lottia gigantea*, and annelid *Capitella teleta*) along with an outgroup (red flour beetle [*Tribolium castaneum])*. Grey bar linking genes represents regions of synteny. Silhouette sources: Phylopic as listed in Acknowledgements and 91–94. Arrows show direction of transcription where known. B, Phylogeny of *P. maximus* Hox and Parahox genes alongside those of known homology from previous work [95, 96] inferred using MrBayes (MrBayes, RRID:SCR_012067) [97] under the Jones model (1,000,000 generations, with 25% discarded as “burn-in”) from a MAFFT alignment under the L-INS-I model [98]. Numbers at base of nodes are posterior probabilities, shown to 2 significant figures. Branches are coloured by gene.

*P. maximus* possesses a single Hox cluster spanning nearly 1.73 Mb (from 28,829,013 to 30,558,725 bp) on scaffold HiC_scaffold_2_arrow_ctg1 (Fig.   4A). It also features a single Parahox cluster on scaffold HiC_scaffold_5_arrow_ctg1. The complex, like that of *M. yessoensis* [22], is stereotypical. This evidence, along with a lack of any obvious signal in our *k-*mer plots (Fig. 2) or previous karyotypic work [52], suggests that no WGD has taken place, although this possibility cannot be completely excluded.

### Immunity to neurotoxins

Bivalves are known to accumulate a number of toxins derived from phytoplankton, and human ingestion of contaminated bivalves can result in 5 known syndromes: ASP caused by DA, PSP from STX, diarrhetic shellfish poisoning from okadaic acid and analogues, neurotoxic shellfish poisoning caused by brevetoxin and analogues, and azaspiracid shellfish poisoning from azaspiracid [16]. Adult *P. maximus* are relatively immune to STX and DA and, as such, may be vectors for the syndromes PSP and ASP, which are of the greatest concern to human health [80, 81].

STX and brevetoxin are neurotoxins that bind to the voltage-gated sodium channel, blocking the passage of nerve impulses [83]. Previous studies have shown that genetic mutations within the sodium channel gene, *Neuron Navigator 1* (*Nav1*), confer immunity in taxa that accumulate STX (e.g., the soft-shell clam *Mya arenaria* [84], scallop *Azumapecten farreri* [21], and copepods *Calanus finmarchicus* and *Acartia hudsonica* [85]) or other similar-acting neurotoxins such as tetrodotoxin (TTX) (e.g., pufferfish, *Tetraodon nigroviridis* and *Takifugu rubripes*; salamanders [86–89]; and the venomous blue-ringed octopus [90]).

**Figure 5:**
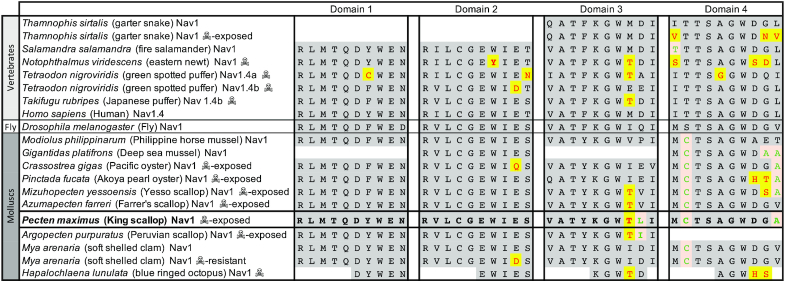
Domain alignments (generated using MAFFT using the E-INS-I model [98]) of the sodium channel *Nav1* showing residues (text in red, highlighted in yellow) implicated in resistance to the neurotoxins tetrodotoxin (TTX) and saxitoxins (STX). Species of vertebrate and mollusc known to be resistant to TTX or STX [86–89] are shown alongside species and sub-populations with no resistance to these toxins. Species (and sub-populations) that produce or accumulate these toxins with little or no ill effect are marked with a skull-and-crossbones. *Pecten maximus* (bold text) shares a thymine residue in domain 3 known to confer neurotoxin resistance in several other species. It also has a number of residues (shown in green text with amber background) in Domains 3 and 4, which are either unique to *P. maximus* or shared with other resistant shellfish, but not seen in other species. These residues are good candidates for testing for a functional role in resistance in the future.

The *P. maximus Nav1* gene possesses the expected canonical domain structure observed in other taxa. Furthermore, it possesses the characteristic thymine residue in Domain 3 (Fig. 5, position 1,425 in reference to rat sodium channel IIA), also described in the other 2 scallop species sequenced so far, which has been shown to confer resistance to these toxins in pufferfish, copepods, and the venomous blue-ringed octopus [85–87]. It does not, however, have the E945D mutation seen in the softshell clam *M. arenaria* and some pufferfish, which experimental evidence suggests also confers resistance [84], nor the D1663H or G1664S mutations in the blue-ringed octopus [90]. Instead, it has 1 novel and 2 ancestrally shared changes (shared with scallops and other bivalves) that may be of interest in studying alternative means of resistance in this molecule.

Unlike STX and TTX, DA does not directly target sodium channels; instead it mimics glutamate and binds preferentially to glutamate receptors including N-methyl-D-aspartate (NDMA), kainate, and α-amino-3-hydroxy-5-methyl-4-isoxazolepropionic acid (AMPA) receptors, leading to elevated levels of intracellular calcium and potentially, calcium toxicity [9, 13]. A recent study, however, has shown that extracellular sodium concentration plays a crucial role in excitotoxicity of DA [99], suggesting that mutations that we observe at *Nav1* may also confer a degree of immunity to DA in *P. maximus*. This has ramifications for the study of neurotoxin resilience and prevalence in the increasingly important commercially fished populations of *P. maximus*.

## Conclusions

The genome of *Pecten maximus* presented here is a well-assembled and annotated resource that will be of utility to a wide range of investigations in scallop, bivalve, and molluscan biology. It is, to date, the best-scaffolded genome available for bivalves, despite the heterozygosity seen in this clade. Given that this assembly is based on state-of-the-art long-range data and has undergone structural verification, this resource will be key for comparative analysis of structural variation and long-range synteny. The curated gene set of this species exhibits little loss compared to other sequenced bivalve species and possesses numerous duplicated genes, which have contributed to the largest gene set observed to date in molluscs. The genes are well annotated, with 88.2% of our high-confidence gene set mapped to a known gene. This genome has already yielded a range of insights into the biology of *P. maximus* and will provide a basis for investigations into fields such as physiology, neurotoxicology, population genetics, and shell formation for many years to come.

## Availability of Supporting Data and Materials

The *Pecten maximus* xPecMax1.1 assembly is available at NCBI under the accession GCA_902652985.1. The data sets supporting the results of this article are available from FigShare [40], GigaDB [41], and also via the DNA Zoo website [55].

## Additional Files

**Supplementary File 1:** Read quality assessment, FastQC/NanoComp

**Supplementary File 2:** Additional Blobplot plots and data, including those separated by phylum/superkingdom

**Supplementary File 3:** ReadsPerGene files output by STAR

**Supplementary File 4:** BLAST annotations, *Pecten maximus* gene models

**Supplementary File 5:** KEGG-KAAS annotations, *Pecten maximus* gene models

giaa037_GIGA-D-20-00019_Original_Submission

giaa037_GIGA-D-20-00019_Revision_1

giaa037_Response_to_Reviewer_Comments_Original_Submission

giaa037_Reviewer_1_Report_Original_Submission Roger Huerlimann -- 2/5/2020 Reviewed

giaa037_Reviewer_2_Report_Original_Submission Takeshi Takeuchi, Ph.D -- 2/6/2020 Reviewed

giaa037_Reviewer_2_Report_Revision_1 Takeshi Takeuchi, Ph.D -- 3/2/2020 Reviewed

giaa037_Reviewer_3_Report_Original_Submission Yi-Jyun Luo -- 2/10/2020 Reviewed

giaa037_Reviewer_3_Report_Revision_1 Yi-Jyun Luo -- 3/9/2020 Reviewed

giaa037_Supplemental_Files

## Abbreviations

AMPA: α-amino-3-hydroxy-5-methyl-4-isoxazolepropionic acid receptors; ASP: amnesiac shellfish poisoning; BLAST: Basic Local Alignment Search Tool; bp: base pairs; BUSCO: Benchmarking Universal Single Copy Orthologs; DA: domoic acid; Gb: gigabase pairs; GC: guanine-cytosine; KAAS: KEGG Automatic Annotation Server; KEGG: Kyoto Encyclopedia of Genes and Genomes; LINE: long interspersed nuclear element; LTR: long terminal repeat; MAFFT: Multiple Alignment using Fast Fourier Transform; Mb: megabase pairs; MIR: mammalian wide interspersed repeat; NDMA: N-methyl-D-aspartate receptors; NIH: National Institutes of Health; PacBio: Pacific Biosciences; PST: paralytic shellfish toxin; RNAseq: RNA sequencing; STX: saxitoxin; SINE: short interspersed nuclear element; TTX: tetrodotoxin; UTR: untranslated region; WGD: whole-genome duplication.

## Competing Interests

The authors declare that they have no competing interests.

## Funding

This work was performed as part of the Wellcome Sanger Institute 25 Genomes Project. Work on this manuscript was performed using funds from NHM DIF (SDR17012) to S.T.W. N.J.K. was supported by an H2020 MSCA grant during the conception of this study and thus this project received funding from the European Union's Horizon 2020 research and innovation programme under the Marie Sklodowska-Curie grant agreement No. 750,937. S.A.M. is supported by Wellcome grant WT207492. E.L.A. was supported by an NSF Physics Frontiers Center Award (PHY1427654), the Welch Foundation (Q-1866), a USDA Agriculture and Food Research Initiative Grant (2017–05741), an NIH 4D Nucleome Grant (U01HL130010), and an NIH Encyclopedia of DNA Elements Mapping Center Award (UM1HG009375). Publication costs were paid with the support of the Marie Curie Alumni Association. Funding sources had no involvement in the decision to submit for publication.

## Authors' Contributions

S.T.W. conceived of the study, provided the tissue samples, and contributed to the text. N.J.K. performed bioinformatic analyses, drafted the manuscript, and prepared the figures. S.A.M. assembled the draft genome. O.D., A.D.O., D.W., and E.L.A. generated and analysed the Hi-C data as part of the DNA Zoo effort. Y.R. and K.J. contributed to bioinformatic analyses, particularly RNAseq. K.H. led the assembly curation, with J.T. performing contamination checks and removal, Y.S. creating assembly analyses, and S.P. performing manual assembly curation. E.B., C.C., J.D., K.O., J.S., M.S., and A.W. aided with DNA extraction, processing, sequencing, and data delivery. D.M. and K.H. were responsible for project organization. All authors approved the final version of the manuscript.
