# Supplementary material for: The gene-rich genome of the scallop Pecten maximus
Source: Gigascience. 2020 Apr 30;9(5):giaa037. doi: 10.1093/gigascience/giaa037 (PMC7191990; doi:10.1093/gigascience/giaa037)
Supplement: giaa037_Response_to_Reviewer_Comments_Original_Submission [file giaa037_response_to_reviewer_comments_original_submission.pdf]

Author's Response To Reviewer Comments

Close

Dear Dr Zauner,

Re: "The Gene-Rich Genome of the Scallop Pecten maximus"

Many thanks for your constructive and positive feedback on our manuscript. Your suggestions and those of the reviewers have definitely improved our work. All comments and our responses are noted below, and we have uploaded both "changes tracked" and "changes accepted" versions of our text so these changes can be verified. We hope that the revised manuscript is now suitable for publication.

Please note that we were unable to upload our Supplementary Data files but these are available at Figshare, as mentioned in the manuscript, at http://dx.doi.org/10.6084/m9.figshare.10311068.

Many thanks,

Suzanne Williams and Nathan Kenny, on behalf of the authors

Editor comments:

-The reviewers agree that the manuscript presents valuable data, however, they also would like to see some important clarifications regarding the methods, in particular with respect to genome assembly and annotation.

Apologies for missing details in our original submission. We have added specific details to make our methods clearer in response to the reviewer's comments, as listed below.

-Compared to other bivalves, this genome seems to have some unusual characteristics, notably the high number of gene models. I agree with the reviewers that this finding should be scrutinized carefully before the manuscript could be acceptable for publication. I hope you can include some additional validation, as well as more details on the methods, to explore the questions brought up by our reviewers.

We have added a variety of specifics regarding our methodology, as requested by yourself and the reviewers, and as noted in detail below. Additionally, to address the concern regarding the high number of genes, we have performed an additional experiment to verify our gene models. We mapped the results from several previously published, independent RNAseq experiments to our "high confidence" gene models and have added this verification to our manuscript. These results confirm the validity of these gene models, with a high percentage of these genes expressed even in tissue-specific RNA samples. The following text has been added to our work (line 312 onwards):

"To confirm the veracity of these gene models as transcribed genes, we mapped samples from a number of previously sequenced, independent RNAseq experiments to our gene models using STAR 2.7 [66] and the --quantMode GeneCounts option. This records only the reads corresponding to one gene, with no multimappers recorded, and is thus a highly stringent test of transcription. Of our 67,741 curated "high confidence" gene models, 47,159 (69.7%) were transcribed in the novel mantle-specific RNA dataset presented in this paper. From independent samples, 33,553 were transcribed in the mantle of the sole control sample from a previous heat stress experiment [56]. 48,882 expressed in two replicate late veliger controls from an experiment where embryos were exposed to a range of pHs (PRJNA298284) and 39,640 were expressed in MiSeq reads sampled from mixed adductor muscle, hepatopancreas, male & female gonad tissue (PRJEB17629). In total, 57,368 of our 67,741 curated "high confidence" gene models (84.7%) are supported by these independent RNAseq experiments, 54,153 (79.9%) of which were found in samples other than our novel transcriptome. These mapping results have been made available for download as Supplementary File 3. It should be noted that this is likely an underestimate of

transcription, given that multi-mapping reads were discounted from consideration. If additional tissues and life stages were targeted, given the fact that these genes have orthologues in closely related species, it is likely that almost all of our gene models would be found to be expressed.”

Reviewer reports:

-Reviewer #1: The authors present a high-quality assembly of the scallop Pecten maximus. In addition to the basic assembly, the authors have carried out a thorough gene annotation and report a high number of genes, compared to other mollusks. Additionally, the authors investigate the possibility of whole genome duplication and also investigate mutations that lead to an immunity to neurotoxins. I would consider this data note highly relevant for other researchers in the field.
Overall, the manuscript is well written and the research was done in a through manner. The methods are appropriate to fulfill the aims of the study. I especially appreciate the inclusion of specific parameters for many of the analyses used; however, there are a few steps that are not described well enough (see below in detailed comments). Furthermore, there are a few programs that have not been referenced.

Many thanks for these positive and constructive comments. We hope we have addressed your concerns below.

Lastly, the lack of line numbering makes this manuscript difficult to review. I highly recommend for future submissions to include line numbering.

Apologies – these are added

Detailed comments:
Abstract
Findings: Change "Here we report the genome sequencing of this species" to "Here we report the genome assembly of this species"

Changed

Findings Line 3: split the two sentences by removing "and". Starting the new sentence with "Its 3,983 scaffolds…"

Changed

Methods
Control: I would like to see more detail on the DNA extraction and clean up methods.

Additional details added (line 150-153)

Page 7: FastQC needs a proper reference.
Andrews, S. (2010). FastQC: a quality control tool for high throughput sequence data.

Updated

Page 7: How was the genome size estimated? Please add reference if taken from another publication.

Estimated using PacBio reads and Minimap2, as noted page 9. A note to this effect has been added to the text

-Citation 32: wtdbg2 / redbean has now been published. Please update reference from biorxiv to Nature Methods.

Updated

Page 7: I would differentiate between the 10X and HiC scaffolding by calling them "medium range scaffolding" and "long range scaffolding". Also, I assume the HiC scaffolding was done on the 10X scaffolded genome, but this should be made clearer in text. Lastly, it is unclear what "manual curation" entailed.

Added the requested text (lines 178-180). Manual curation with Juicebox allows conflicting evidence from HiC data to be critically evaluated and either split (in case of doubt) or corrected. gEVAL allows identification of errors in assembly, by displaying areas where data disagrees with the assembly. These steps are standard in these processes, and more detail is available from the references provided, but the text has been altered to indicate how this occurs.

Page 8, first line: Again, what does "manually improved" mean?

Please see reply immediately above

Assembly Assessment
Page 9: how was heterozygozity calculated?

Calculated by Genomescope on the basis of kmers – reference added

Gene Prediction and Annotation
Page 10: should be `set to "true"`

Corrected

Page 11: What do you consider "a good hit", purely based on the e-value? I find that sometimes you can get a small partial hit with low query coverage (<10%) and still have "good" e-value.

As the reviewer correctly points out, this will vary, and depend directly on the size of the database (as e values are calculated from that size). We had to balance recovery of true homologues with the removal of spurious matches. We have added a note to the text, Line 299, stating how this value was chosen

"empirically determined "good" hit in the nr database, lenient enough to recover genes from more distantly related species but stringent enough to avoid chance similarity"

Page 11: please keep your decimals consistent, e.g. 1e-9 vs 1.0e-29

Corrected

Page 11: Throughout publication, don't directly refer to figures and tables: "This is comparable to previously published bivalve resources, as can be seen in Table 3" vs. "This is comparable to previously published bivalve resources (Table 3)"

Corrected here and elsewhere – several references to figures corrected.

Please provide proper references for all programs used, e.g. blast and diamond.

Cited throughout as appropriate

Page 11, last paragraph: What are these "automated methods"? How does this blast search differ to the one mentioned above on the same page? Which blast type was used? Which reference database was used and when was it accessed?

Sorry for the confusion, the automated methods are those detailed later in the same paragraph. This has been made clearer by including the word "two" in the first sentence. The blast used was more lenient than that on the previous page (note e value cutoff and tblastn vs blastp) – this has been noted in text. We have added the blast type (tblastn) and noted the date of the version of nr used.

Gene complement and expansion
Pages 12 to 13: The discussion of orthologous genes and how they occur in bivalves is out of my area of expertise; however, based on my understanding of the topic the analysis and conclusions look valid. The authors do rightly caution the reader that these could be the result of incomplete gene prediction in some species.

Thanks for this comment – we hope we have presented the limitations of this fairly.

Figure 2C: This figure is difficult to interpret due its "zoomed out nature: and I'm not sure how much it

is adding to the publication.

Apologies for the scale – in the final text it will be easier to view in larger resolution. It has proven useful to Reviewers below, so we have kept it, although we would be willing to remove it if this is requested by the reviewers.

Tables 1, 3 and 4: Please make sure you keep decimals consistent within each "type" (e.g. Assembly length in table 3, or % of genome in Table 4)).

Table 1: Rounded up one number to make consistent
Table 3: Added .1 to Assembly length for Saccostrea glomerata and .3 to Pecten maximus
Table 4: removed rounding in % of genome

Table 3: there are some issues here with referencing.

Fixed

Reviewer #2: Kenny et al. succeeded to establish a chromosome-level genome assembly of the king scallop Pecten maximus using the Pac Bio long read data for contig assembling, and 10x Chromium and Hi-C for scaffolding. The high-quality genome assembly is valuable to understand the basic biology of the species.
However, I have concerns about the method and result of the gene prediction, showing such a large number of gene models (215,598) compare to other bivalves (30~40k). It is necessary to re-analyze the gene prediction and validate them before publication. Detailed descriptions of methods, specially regarding novel RNA-seq data, are required.
Below I have specific comments and I hope they are helpful to improve the paper.

Many thanks for your constructive and positive comments. With regard to the gene number given above, we feel that the reviewer may have interpreted our unfiltered gene number (215,598) as the final gene number, and has made some conclusions on the basis of that. We have responded to the reviewer's specific points below.

P.4 "Previous studies... and reproduction."
References are needed.

Examples have been inserted

p.5 " Of these resources..."
The authors may want to add a recently published paper of Sinonovacula constricta genome (Ran et al., Mol Ecol Resour. 2019;19:1647-1658).

This has been added

p.8 "...19 pars of chromosomes, in agreement with prior studies[37],..."
A prior study?

Changed to the singular

p.9 "It should be noted that we used Purge Haplotigs..."
It is not clear whether they used Purge Haplotigs to remove redundant sequences from the assembly. If yes, the method should be mentioned in the Genome assembly section.

Apologies - freebayes-polish was used to polish heterozygosity. This has been corrected in text at this point.

P.10 "Gene sequences were predicted... with one novel and several previously published P. maximus RNAseq datasets [47, 48] used for training."
The authors should explain the novel RNA-seq data in detail (e.g. from which tissue(s) RNA was extracted? method for RNA extraction, library preparation, sequencing platform, amount of raw data, assembly software etc.). I checked the supplementary data and found only one fasta file of transcriptome assembly.

We have added additional information to this point of the paper (line 269 onward) noting the requested details. These are copied below for ease of verification.

“The novel dataset was derived from two samples of P. maximus mantle from the same specimen used for gDNA extraction. These were sequenced on an Illumina HiSeq to a depth of 338,910,597 reads. After initial trimming of poor quality sequence and residual adaptors with TrimGalore v0.6 [58], this library was assembled using Trinity v 2.8.4 [59] with all default settings. Following assembly, chimeric, fragmented, or locally misassembled transcripts were filtered using Transrate v1.0.3 [60], where ’good’ transcripts were retained, followed by DETONATE v1.11 with the bowtie2 option [61], where transcripts scoring < 0 were discarded. Transcripts were the clustered using cd-hit-est v 4.8.1 [62] at an identity threshold of 95% (- c 0.95 -n 8 -g 1), and the representative sequence of each cluster was retained.”

How the RNA-seq data was applied for the training? Which software did they use for mapping and training pipeline?

The RNA seq data is used natively by AUGUSTUS using BLAT. We have added a note regarding this (Lines 280-281)

“Training was first performed using the RNAseq datasets noted above, as part of the AUGUSTUS pipeline (incorporating BLAT alignment [63]).”

According to their description, they used the RNA-seq data for training but not for "hints" in gene prediction. I strongly recommend to generate a hint file based on the RNA-seq and apply it to AUGUSTUS in order to improve the gene prediction.

This was done but is not clear from the text. We have made this obvious (line 281/282).

“After training, the resulting hints file was submitted once more to Augustus for prediction, alongside the same mRNA files used for initial training.”

P.10 "215,598 putative genes"
P.11 "final, 67,741, curated set (of genes)"
These numbers are much higher than that of other molluscs, presumably due to false-positive and fragmented gene prediction. The authors discussed that "This number,...is comparable to the number of unigenes in Argopecten irradians (P.11)". However, the draft assembly of the A. irradians is considerably fragmented (the number of scaffold is 217,310 and scaffold N50 is 6.8kb), and therefore the genes might be divided into short and incomplete gene models.
In order to validate the gene models, I would suggest to calculate average length of CDS and number of exons per gene, and compare them to those of other bivalve genomes. In addition, how many gene models are supported by RNA-seq?

We have performed an additional series of experiments to confirm our gene models independently, using prior RNAseq datasets. This is noted in our response to the editor above, but can be seen in the text (line 312 onwards) and is copied below for completeness. We trust that this addresses the concern regarding the veracity of these gene models – transcription is excellent proof of their existence.

“To confirm the veracity of these gene models as transcribed genes, we mapped samples from a number of previously sequenced, independent RNAseq experiments to our gene models using STAR 2.7 [66] and the --quantMode GeneCounts option. This records only the reads corresponding to one gene, with no multimappers recorded, and is thus a highly stringent test of transcription. Of our 67,741 curated “high confidence” gene models, 47,159 (69.7%) were transcribed in the novel mantle-specific RNA dataset presented in this paper. From independent samples, 33,553 genes were transcribed in the mantle of the sole control sample from a previous heat stress experiment [56]. A total of 48,882 genes were expressed in two replicate late veliger controls from an experiment where embryos were exposed to a range of water conditions (varying pH) (PRJNA298284) and 39,640 were expressed in MiSeq reads sampled from mixed adductor muscle, hepatopancreas, male & female gonad tissue (PRJEB17629). In total, 57,368 of our 67,741 curated “high confidence” gene models (84.7%) are supported by these independent RNAseq experiments, 54,153 (79.9%) of which were found in samples other than our novel transcriptome. These mapping results have been made available for download as Supplementary File 3. It should be noted that this is likely an underestimate of transcription, given that multi-mapping reads were discounted from consideration. If additional tissues and life stages were targeted, given the fact that these genes have known orthologues in closely related species (see Orthofinder2 results above), it

is likely that almost all of our gene models would be found to be expressed.”

The suggestion to calculate average number of exons proved to be very informative - thank you for this! We have added text to this regard on line 335-339, copied below:

“The 84,866 transcripts in our high confidence gene set (some genes possess more than one transcript), have an average of 5 exons. This is fewer than that seen in M. yessoensis, (7 exons on average) or P. fucata (6 on average) [Table S8, 22]. This may indicate a degree of fragmentation in our gene models (although that is not observed empirically), or alternatively, that some of the genes in our gene models have been copied via retrotransposition and lack introns, which would lower the average exon number and contribute to the high number of genes seen in this species..”

P.11 "seven previously published bivalves"
Which seven species?

Added names in text at this point

Figure 4
How did they conduct multiple alignment for the molecular phylogeny?

MAFFT – added along with citation to legend

Figure 5
Again, how did they make the multiple alignment?

MAFFT – added along with citation to legend

Reviewer #3: The authors presented a high-quality scallop genome of Pecten maximus. Using PacBio long reads followed by scaffolding with 10x Chromium and Hi-C, they generated the genome assembly of the chromosomal level. After gene annotation, the authors analyzed the Hox gene cluster and neurotoxins. The sequencing method is state-of-the-art, and the manuscript is well presented. I have comments mostly on their genome assembly and gene annotation methods as follows.

Many thanks for your supportive and constructive comments

Major comments:
1. There are 67,741 gene models (even after filtering) found in the P. maximus genome. This number is very high among animals. I noticed that the authors performed gene prediction based on a non-masked genome. Would it introduce prediction errors? To my knowledge, people usually predict genes using a masked genome. That is to avoid the misprediction of genes from repetitive elements. From my experience, using the gene prediction program, Augustus, with UTR setting is not very good for non-model species. I am concerning that gene annotation with UTR prediction might be troublesome. It is particularly the case when the authors got 215,598 putative genes.

Using a masked genome is itself a potential cause of error in predicted sequences – any genes that overlap at their margins with repetitive sequence (even on the other strand) will be incorrectly truncated, resulting in artifactually shortened gene models. We therefore predicted genes with our unmasked assembly, then secondarily removed any genes from repetitive elements from our high confidence gene set (see line 302… “However, we then removed from this combined total any genes which had a match within our identified repetitive elements (13,374 genes….”).

For gene model number, please note our extra confirmatory experiment, noted in the response to the editor at the top of this document.

UTR prediction is indeed difficult, although this is problematic for correct recognition of UTRs, rather than the coding sequences. We have added the following note to our manuscript (Lines 285-287):

“Please note that UTR prediction with AUGUSTUS is imperfect in non-model organisms, and UTR regions provided here are current best estimates, and would benefit from full length RNA sequencing (e.g. Isoseq, on the PacBio platform).”

2. Following the first comment, how could the authors make sure that they have a haplotype genome assembly [editor's note: I assume the reviewer means "haploid genome assembly"] using the long-read approach? Is there a step that the authors can assure that two highly variable allele scaffolds can be collapsed into one? This possible redundancy is a particular concern when the species has high heterozygosity. Is it possible that 67,741 gene models predicted in the P. maximus genome is due to having a redundant diploid genome?

We did not make the removal of heterozygous regions clear enough to the reader, but have scrubbed this comprehensively from this assembly. Heterozygosity was removed using freebayes-polish (which incorporates bcftools consensus). The high contiguity and excellent scaffolding of this resource, coupled with this approach, makes it unlikely that any large degree of heterozygosity remains, although it is possible that small fragments with extreme heterozygosity.

Furthermore, as 92% of the genome is in chromosomal level scaffolds, 8% of the genome would be the absolute maximum possible level of heterozygosity present (although this is highly unlikely), and could not explain the higher gene number, even if this was the case.

We have added ", and no detectable heterozygosity will remain" to line 243 to make the role of freebayes-polish more clear to the reader.

3. Assembly Assessment: What is the primary reason that P. maximus is much larger than Crassostrea gigas and Lottia gigantea? If that is not due to the repeats, what about the intergenic region or intron size among these species?

The C value paradox (genome size) is a difficult problem, and is not entirely understood. It should be noted that Crassostrea gigas and Lottia gigantea were chosen for sequencing partially as they have small genomes – it is these species that are unusual, not Pecten maximus.

To acknowledge these possible causes, we have added the following sentence to this work (line 225): "The reasons for these differences in genome size are at present unclear, but may include gene duplications, repetitive element expansions and, in some cases, whole genome duplications [50]".

4. For those scaffolds with blast similarity to Proteobacteria, do all the genes on those scaffolds have blast hits to Proteobacteria genes? Panel C in Figure 2 is difficult to see, especially for the color code. Maybe consider to zoom-in a bit and adjust data visualization (e.g., circle size). I definitely can see that some circles have high GC (>0.4) and coverage (>100). Are those possible contamination (their colors are not easily visible)?

Not all genes on these scaffolds have hits to Proteobacteria, so this could be chance similarity. Changes to circle size and zooming omitted useful information. To make these plots easier to view in detail (and to provide per-Phylum and per-Superkingdom information) we have additionally provided these and additional plots as Supplementary File 2, and changed the text to indicate this.

5. Could the authors explain why P. maximus has 518 species-specific orthogroups? This number seems to be unreasonably high compared to those in other molluscs. Similar concern for the unassigned genes (158,024 genes in P. maximus compared to 2,000-7,000 in other species).

This number is high as it is derived from the full (uncurated) gene set. These numbers are likely repetitive sequence – the following has been added to the text (line 376):

"but they may be derived from repetitive content, as the unfiltered P. maximus gene set was used as the basis of this comparison."

6. Did the authors perform any test to assess whether P. maximus has the whole-genome duplication (WGD)? Only one example of the Hox gene cluster is not convincing to exclude the possibility of WGD.

We cannot completely exclude the possibility of a WGD event at some point in the ancestry of Pecten maximus. However, there is no evidence of one in our kmer plots, in previous karyotypic work, or (as noted) in the Hox or Parahox clusters. We have made this more obvious, adding the following (line 422):

"This evidence, along with a lack of any obvious signal in our k-mer plots (Fig.2) or previous karyotypic work [38] suggests that no WGD has taken place, although this possibility cannot be completely

excluded.”

Minor comments:
1. There are some small typos and format issues. But without line numbers labeled, it is difficult to point them out. The authors should add line numbers for the revised version.

Added lines – hopefully we have dealt with these problems, both as raised by other reviewers and by spotting them ourselves.

2. Repeat elements -> "Repetitive elements" for consistency.

Changed in two locations

3. c.f. -> "cf."

Corrected

Close
