# Supplementary material for: The gene-rich genome of the scallop Pecten maximus
Source: Gigascience. 2020 Apr 30;9(5):giaa037. doi: 10.1093/gigascience/giaa037 (PMC7191990; doi:10.1093/gigascience/giaa037)
Supplement: giaa037_Reviewer_1_Report_Original_Submission — Roger Huerlimann -- 2/5/2020 Reviewed [file giaa037_reviewer_1_report_original_submission.pdf]

Reviewer Report

Title: The Gene-Rich Genome of the Scallop Pecten maximus

Version: Original Submission **Date:** 2/5/2020

Reviewer name: Roger Huerlimann

Reviewer Comments to Author:

The authors present a high-quality assembly of the scallop Pecten maximus. In addition to the basic assembly, the authors have carried out a thorough gene annotation and report a high number of genes, compared to other mollusks. Additionally, the authors investigate the possibility of whole genome duplication and also investigate mutations that lead to an immunity to neurotoxins. I would consider this data note highly relevant for other researchers in the field.

Overall, the manuscript is well written and the research was done in a through manner. The methods are appropriate to fulfill the aims of the study. I especially appreciate the inclusion of specific parameters for many of the analyses used; however, there are a few steps that are not described well enough (see below in detailed comments). Furthermore, there are a few programs that have not been referenced.

Lastly, the lack of line numbering makes this manuscript difficult to review. I highly recommend for future submissions to include line numbering.

Detailed comments:

Abstract

Findings: Change "Here we report the genome sequencing of this species" to "Here we report the genome assembly of this species"

Findings Line 3: split the two sentences by removing "and". Starting the new sentence with "Its 3,983 scaffolds…"

Methods

Control: I would like to see more detail on the DNA extraction and clean up methods.

Page 7: FastQC needs a proper reference.

Andrews, S. (2010). FastQC: a quality control tool for high throughput sequence data.

Page 7: How was the genome size estimated? Please add reference if taken from another publication.

Citation 32: wtdbg2 / redbean has now been published. Please update reference from biorxiv to Nature Methods.

Page 7: I would differentiate between the 10X and HiC scaffolding by calling them "medium range scaffolding" and "long range scaffolding". Also, I assume the HiC scaffolding was done on the 10X scaffolded genome, but this should be made clearer in text. Lastly, it is unclear what "manual curation" entailed.

Page 8, first line: Again, what does "manually improved" mean?

Assembly Assessment

Page 9: how was heterozygozity calculated?

Gene Prediction and Annotation

Page 10: should be `set to "true"`
Page 11: What do you consider "a good hit", purely based on the e-value? I find that sometimes you can get a small partial hit with low query coverage (<10%) and still have "good" e-value.
Page 11: please keep your decimals consistent, e.g. 1e-9 vs 1.0e-29
Page 11: Throughout publication, don't directly refer to figures and tables: "This is comparable to previously published bivalve resources, as can be seen in Table 3" vs. "This is comparable to previously published bivalve resources (Table 3)"
Please provide proper references for all programs used, e.g. blast and diamond.
Page 11, last paragraph: What are these "automated methods"? How does this blast search differ to the one mentioned above on the same page? Which blast type was used? Which reference database was used and when was it accessed?
Gene complement and expansion
Pages 12 to 13: The discussion of orthologous genes and how they occur in bivalves is out of my area of expertise; however, based on my understanding of the topic the analysis and conclusions look valid. The authors do rightly caution the reader that these could be the result of incomplete gene prediction in some species.
Figure 2C: This figure is difficult to interpret due its "zoomed out nature: and I'm not sure how much it is adding to the publication.
Tables 1, 3 and 4: Please make sure you keep decimals consistent within each "type" (e.g. Assembly length in table 3, or % of genome in Table 4)).
Table 3: there are some issues here with referencing.

Level of Interest

Please indicate how interesting you found the manuscript: Choose an item.

Quality of Written English

Please indicate the quality of language in the manuscript: Choose an item.

Declaration of Competing Interests

Please complete a declaration of competing interests, considering the following questions:

- Have you in the past five years received reimbursements, fees, funding, or salary from an organisation that may in any way gain or lose financially from the publication of this manuscript, either now or in the future?
- Do you hold any stocks or shares in an organisation that may in any way gain or lose financially from the publication of this manuscript, either now or in the future?
- Do you hold or are you currently applying for any patents relating to the content of the manuscript?

- Have you received reimbursements, fees, funding, or salary from an organization that holds or has applied for patents relating to the content of the manuscript?
- Do you have any other financial competing interests?
- Do you have any non-financial competing interests in relation to this paper?

If you can answer no to all of the above, write 'I declare that I have no competing interests' below. If your reply is yes to any, please give details below.

I declare that I have no competing interests

I agree to the open peer review policy of the journal. I understand that my name will be included on my report to the authors and, if the manuscript is accepted for publication, my named report including any attachments I upload will be posted on the website along with the authors' responses. I agree for my report to be made available under an Open Access Creative Commons CC-BY license (http://creativecommons.org/licenses/by/4.0/). I understand that any comments which I do not wish to be included in my named report can be included as confidential comments to the editors, which will not be published.

Choose an item.

To further support our reviewers, we have joined with Publons, where you can gain additional credit to further highlight your hard work (see: https://publons.com/journal/530/gigascience). On publication of this paper, your review will be automatically added to Publons, you can then choose whether or not to claim your Publons credit. I understand this statement.

Yes Choose an item.
