# Supplementary material for: The gene-rich genome of the scallop Pecten maximus
Source: Gigascience. 2020 Apr 30;9(5):giaa037. doi: 10.1093/gigascience/giaa037 (PMC7191990; doi:10.1093/gigascience/giaa037)
Supplement: giaa037_Reviewer_2_Report_Original_Submission — Takeshi Takeuchi, Ph.D -- 2/6/2020 Reviewed [file giaa037_reviewer_2_report_original_submission.pdf]

Reviewer Report

Title: The Gene-Rich Genome of the Scallop Pecten maximus

Version: Original Submission **Date:** 2/6/2020

Reviewer name: Takeshi Takeuchi, Ph.D

Reviewer Comments to Author:

Kenny et al. succeeded to establish a chromosome-level genome assembly of the king scallop Pecten maximus using the Pac Bio long read data for contig assembling, and 10x Chromium and Hi-C for scaffolding. The high-quality genome assembly is valuable to understand the basic biology of the species.
However, I have concerns about the method and result of the gene prediction, showing such a large number of gene models (215,598) compare to other bivalves (30~40k). It is necessary to re-analyze the gene prediction and validate them before publication. Detailed descriptions of methods, specially regarding novel RNA-seq data, are required.
Below I have specific comments and I hope they are helpful to improve the paper.
P.4 "Previous studies... and reproduction."
References are needed.
p.5 " Of these resources..."
The authors may want to add a recently published paper of Sinonovacula constricta genome (Ran et al., Mol Ecol Resour. 2019;19:1647-1658).
p.8 "...19 pars of chromosomes, in agreement with prior studies[37],..."
A prior study?
p.9 "It should be noted that we used Purge Haplotigs..."
It is not clear whether they used Purge Haplotigs to remove redundant sequences from the assembly. If yes, the method should be mentioned in the Genome assembly section.
P.10 "Gene sequences were predicted... with one novel and several previously published P. maximus RNAseq datasets [47, 48] used for training."
The authors should explain the novel RNA-seq data in detail (e.g. from which tissue(s) RNA was extracted? method for RNA extraction, library preparation, sequencing platform, amount of raw data, assembly software etc.). I checked the supplementary data and found only one fasta file of transcriptome assembly.
How the RNA-seq data was applied for the training? Which software did they use for mapping and training pipeline?
According to their description, they used the RNA-seq data for training but not for "hints" in gene prediction. I strongly recommend to generate a hint file based on the RNA-seq and apply it to AUGUSTUS in order to improve the gene prediction.
P.10 "215,598 putative genes"
P.11 "final, 67,741, curated set (of genes)"
These numbers are much higher than that of other molluscs, presumably due to false-positive and

fragmented gene prediction. The authors discussed that "This number,...is comparable to the number of unigenes in Argopecten irradians (P.11)". However, the draft assembly of the A. irradians is considerably fragmented (the number of scaffold is 217,310 and scaffold N50 is 6.8kb), and therefore the genes might be divided into short and incomplete gene models.
In order to validate the gene models, I would suggest to calculate average length of CDS and number of exons per gene, and compare them to those of other bivalve genomes. In addition, how many gene models are supported by RNA-seq?
P.11 "seven previously published bivalves"
Which seven species?
Figure 4
How did they conduct multiple alignment for the molecular phylogeny?
Figure 5
Again, how did they make the multiple alignment?

Level of Interest

Please indicate how interesting you found the manuscript: Choose an item.

Quality of Written English

Please indicate the quality of language in the manuscript: Choose an item.

Declaration of Competing Interests

Please complete a declaration of competing interests, considering the following questions:

- Have you in the past five years received reimbursements, fees, funding, or salary from an organisation that may in any way gain or lose financially from the publication of this manuscript, either now or in the future?
- Do you hold any stocks or shares in an organisation that may in any way gain or lose financially from the publication of this manuscript, either now or in the future?
- Do you hold or are you currently applying for any patents relating to the content of the manuscript?
- Have you received reimbursements, fees, funding, or salary from an organization that holds or has applied for patents relating to the content of the manuscript?
- Do you have any other financial competing interests?
- Do you have any non-financial competing interests in relation to this paper?

If you can answer no to all of the above, write 'I declare that I have no competing interests' below. If your reply is yes to any, please give details below.

I declare that I have no competing interests.

I agree to the open peer review policy of the journal. I understand that my name will be included on my report to the authors and, if the manuscript is accepted for publication, my named report including any attachments I upload will be posted on the website along with the authors' responses. I agree for my report to be made available under an Open Access Creative Commons CC-BY license (http://creativecommons.org/licenses/by/4.0/). I understand that any comments which I do not wish to be included in my named report can be included as confidential comments to the editors, which will not be published.

Choose an item.

To further support our reviewers, we have joined with Publons, where you can gain additional credit to further highlight your hard work (see: https://publons.com/journal/530/gigascience). On publication of this paper, your review will be automatically added to Publons, you can then choose whether or not to claim your Publons credit. I understand this statement.

Yes Choose an item.
