# Supplementary material for: The gene-rich genome of the scallop Pecten maximus
Source: Gigascience. 2020 Apr 30;9(5):giaa037. doi: 10.1093/gigascience/giaa037 (PMC7191990; doi:10.1093/gigascience/giaa037)
Supplement: giaa037_Reviewer_3_Report_Original_Submission — Yi-Jyun Luo -- 2/10/2020 Reviewed [file giaa037_reviewer_3_report_original_submission.pdf]

Reviewer Report

Title: The Gene-Rich Genome of the Scallop Pecten maximus

Version: Original Submission **Date:** 2/10/2020

Reviewer name: Yi-Jyun Luo

Reviewer Comments to Author:

The authors presented a high-quality scallop genome of Pecten maximus. Using PacBio long reads followed by scaffolding with 10x Chromium and Hi-C, they generated the genome assembly of the chromosomal level. After gene annotation, the authors analyzed the Hox gene cluster and neurotoxins. The sequencing method is state-of-the-art, and the manuscript is well presented. I have comments mostly on their genome assembly and gene annotation methods as follows.

Major comments:

1. There are 67,741 gene models (even after filtering) found in the P. maximus genome. This number is very high among animals. I noticed that the authors performed gene prediction based on a non-masked genome. Would it introduce prediction errors? To my knowledge, people usually predict genes using a masked genome. That is to avoid the misprediction of genes from repetitive elements. From my experience, using the gene prediction program, Augustus, with UTR setting is not very good for non-model species. I am concerning that gene annotation with UTR prediction might be troublesome. It is particularly the case when the authors got 215,598 putative genes.
2. Following the first comment, how could the authors make sure that they have a haplotype genome assembly using the long-read approach? Is there a step that the authors can assure that two highly variable allele scaffolds can be collapsed into one? This possible redundancy is a particular concern when the species has high heterozygosity. Is it possible that 67,741 gene models predicted in the P. maximus genome is due to having a redundant diploid genome?
3. Assembly Assessment: What is the primary reason that P. maximus is much larger than Crassostrea gigas and Lottia gigantea? If that is not due to the repeats, what about the intergenic region or intron size among these species?
4. For those scaffolds with blast similarity to Proteobacteria, do all the genes on those scaffolds have blast hits to Proteobacteria genes? Panel C in Figure 2 is difficult to see, especially for the color code. Maybe consider to zoom-in a bit and adjust data visualization (e.g., circle size). I definitely can see that some circles have high GC (>0.4) and coverage (>100). Are those possible contamination (their colors are not easily visible)?
5. Could the authors explain why P. maximus has 518 species-specific orthogroups? This number seems to be unreasonably high compared to those in other molluscs. Similar concern for the unassigned genes (158,024 genes in P. maximus compared to 2,000-7,000 in other species).
6. Did the authors perform any test to assess whether P. maximus has the whole-genome duplication (WGD)? Only one example of the Hox gene cluster is not convincing to exclude the possibility of WGD.

Minor comments:

1. There are some small typos and format issues. But without line numbers labeled, it is difficult to

point them out. The authors should add line numbers for the revised version.
2. Repeat elements -> "Repetitive elements" for consistency.
3. c.f. -> "cf."

Level of Interest

Please indicate how interesting you found the manuscript: Choose an item.

Quality of Written English

Please indicate the quality of language in the manuscript: Choose an item.

Declaration of Competing Interests

Please complete a declaration of competing interests, considering the following questions:

- Have you in the past five years received reimbursements, fees, funding, or salary from an organisation that may in any way gain or lose financially from the publication of this manuscript, either now or in the future?
- Do you hold any stocks or shares in an organisation that may in any way gain or lose financially from the publication of this manuscript, either now or in the future?
- Do you hold or are you currently applying for any patents relating to the content of the manuscript?
- Have you received reimbursements, fees, funding, or salary from an organization that holds or has applied for patents relating to the content of the manuscript?
- Do you have any other financial competing interests?
- Do you have any non-financial competing interests in relation to this paper?

If you can answer no to all of the above, write 'I declare that I have no competing interests' below. If your reply is yes to any, please give details below.

I declare that I have no competing interests.

I agree to the open peer review policy of the journal. I understand that my name will be included on my report to the authors and, if the manuscript is accepted for publication, my named report including any attachments I upload will be posted on the website along with the authors' responses. I agree for my report to be made available under an Open Access Creative Commons CC-BY license (http://creativecommons.org/licenses/by/4.0/). I understand that any comments which I do not wish to be included in my named report can be included as confidential comments to the editors, which will not be published.

Choose an item.

To further support our reviewers, we have joined with Publons, where you can gain additional credit to further highlight your hard work (see: https://publons.com/journal/530/gigascience). On publication of this paper, your review will be automatically added to Publons, you can then choose whether or not to claim your Publons credit. I understand this statement.

Yes Choose an item.
