# Supplementary material for: The gene-rich genome of the scallop Pecten maximus
Source: Gigascience. 2020 Apr 30;9(5):giaa037. doi: 10.1093/gigascience/giaa037 (PMC7191990; doi:10.1093/gigascience/giaa037)
Supplement: giaa037_Supplemental_Files [file giaa037_supplemental_files.zip › SupplementaryFile1.zip!/SupplementaryFile1/NanoCompPacBioReads/NanoComp_number_of_reads.pdf]

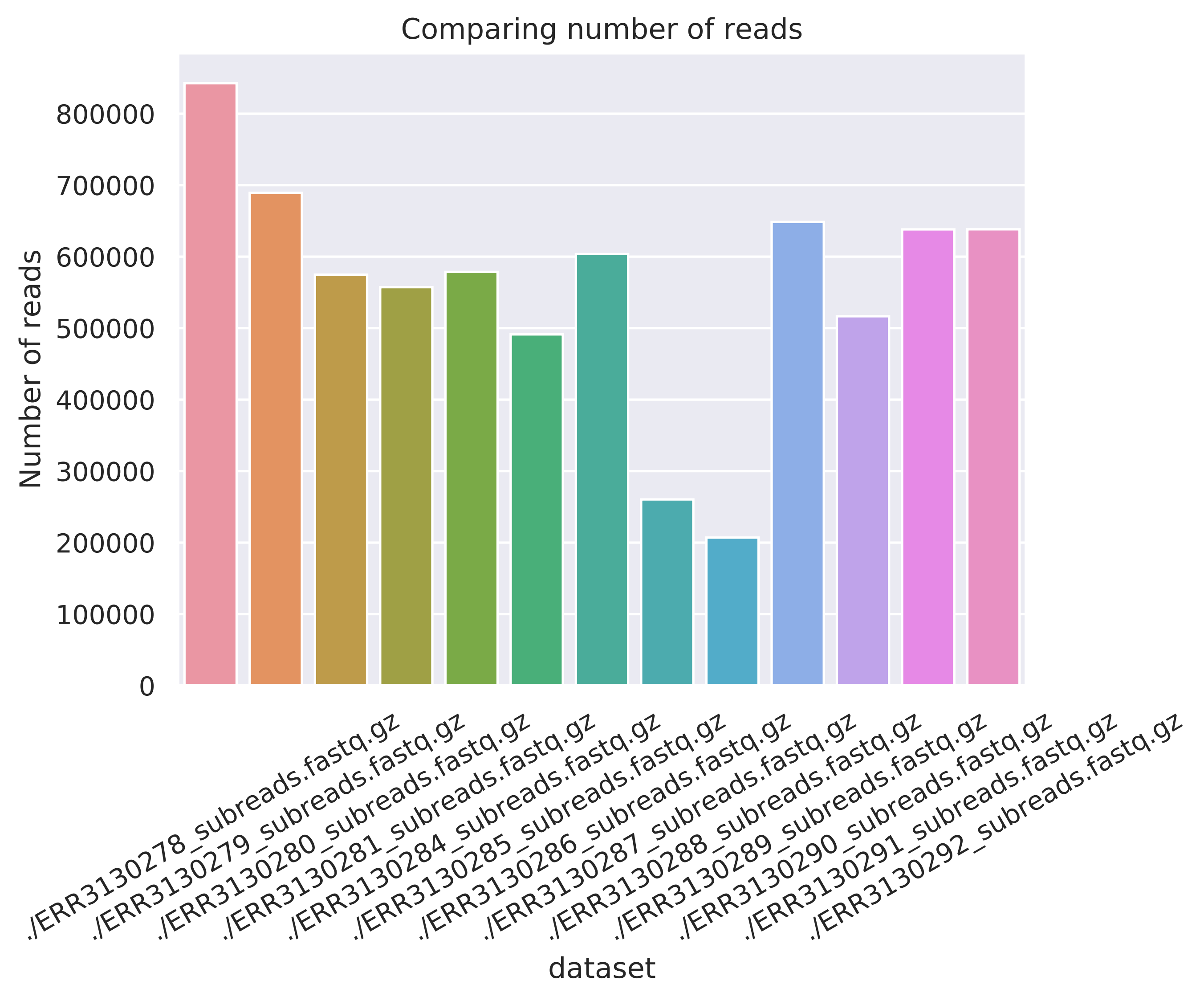

Comparing number of reads
Number of reads
800000
700000
600000
500000
400000
300000
200000
100000
0
./ERR3130278_subreads.fastq.gz
./ERR3130279_subreads.fastq.gz
./ERR3130280_subreads.fastq.gz
./ERR3130281_subreads.fastq.gz
./ERR3130284_subreads.fastq.gz
./ERR3130285_subreads.fastq.gz
./ERR3130286_subreads.fastq.gz
./ERR3130287_subreads.fastq.gz
./ERR3130288_subreads.fastq.gz
./ERR3130289_subreads.fastq.gz
./ERR3130290_subreads.fastq.gz
./ERR3130291_subreads.fastq.gz
./ERR3130292_subreads.fastq.gz
dataset
