# Supplementary material for: The gene-rich genome of the scallop Pecten maximus
Source: Gigascience. 2020 Apr 30;9(5):giaa037. doi: 10.1093/gigascience/giaa037 (PMC7191990; doi:10.1093/gigascience/giaa037)
Supplement: giaa037_Supplemental_Files [file giaa037_supplemental_files.zip › SupplementaryFile1.zip!/SupplementaryFile1/NanoCompPacBioReads/NanoComp_log_length.pdf]

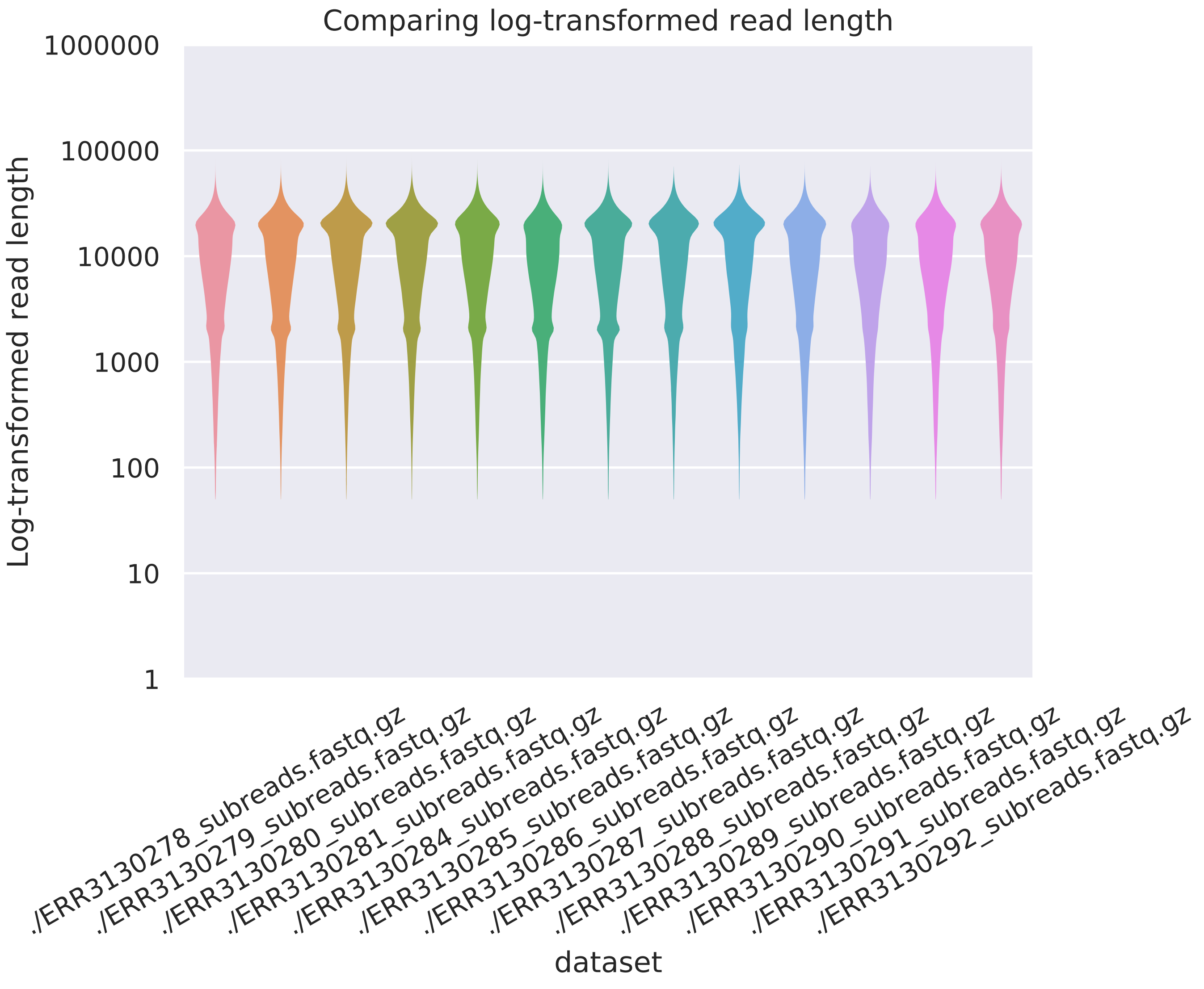
Comparing log-transformed read length
Log-transformed read length
1000000
100000
10000
1000
100
10
1
./ERR3130278_subreads.fastq.gz
./ERR3130279_subreads.fastq.gz
./ERR3130280_subreads.fastq.gz
./ERR3130281_subreads.fastq.gz
./ERR3130284_subreads.fastq.gz
./ERR3130285_subreads.fastq.gz
./ERR3130286_subreads.fastq.gz
./ERR3130287_subreads.fastq.gz
./ERR3130288_subreads.fastq.gz
./ERR3130289_subreads.fastq.gz
./ERR3130290_subreads.fastq.gz
./ERR3130291_subreads.fastq.gz
./ERR3130292_subreads.fastq.gz
dataset
