# Supplementary material for: The gene-rich genome of the scallop Pecten maximus
Source: Gigascience. 2020 Apr 30;9(5):giaa037. doi: 10.1093/gigascience/giaa037 (PMC7191990; doi:10.1093/gigascience/giaa037)
Supplement: giaa037_Supplemental_Files [file giaa037_supplemental_files.zip › SupplementaryFile1.zip!/SupplementaryFile1/NanoCompPacBioReads/NanoComp_total_throughput.pdf]

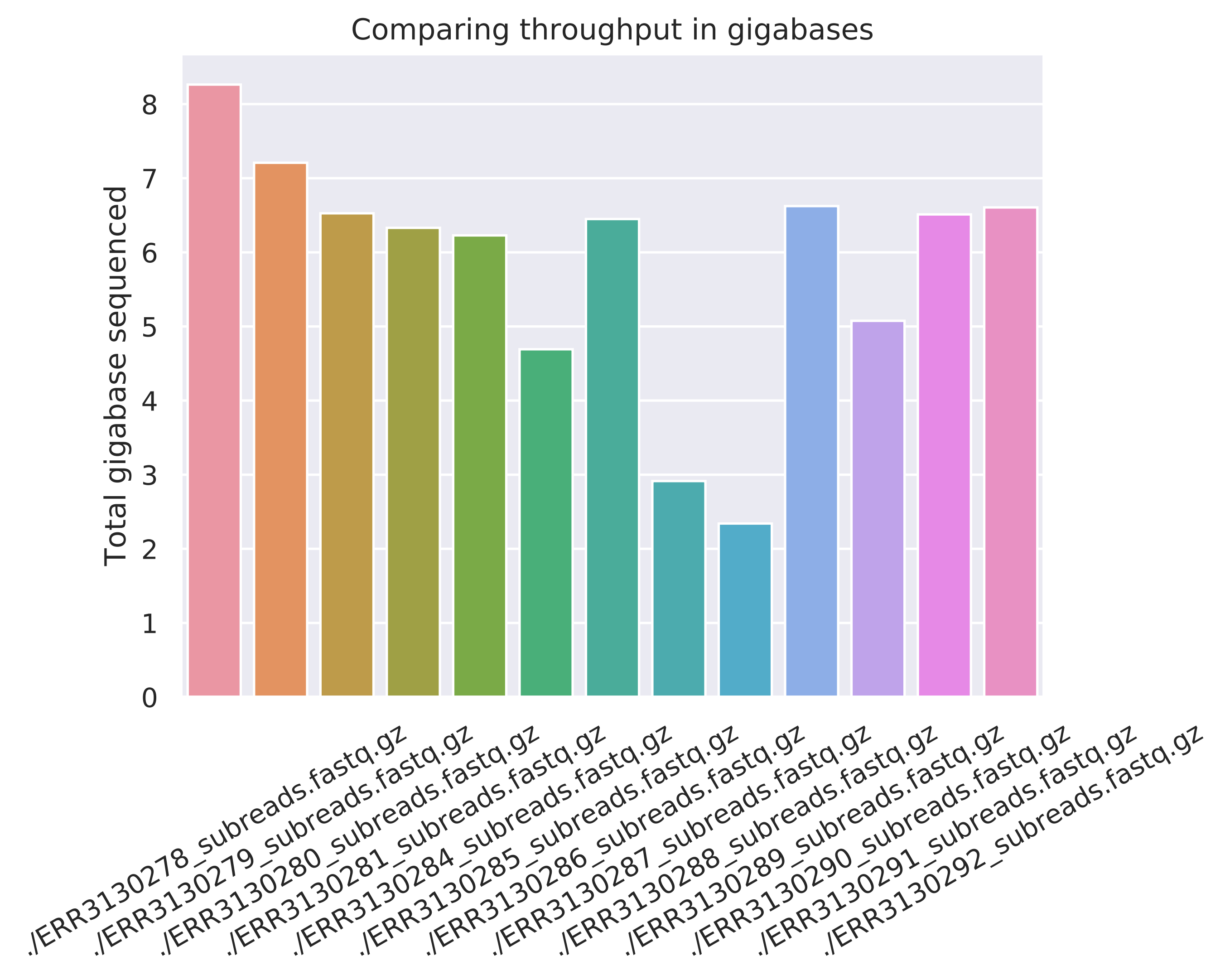

Comparing throughput in gigabases
Total gigabase sequenced
0
1
2
3
4
5
6
7
8
./ERR3130278_subreads.fastq.gz
./ERR3130279_subreads.fastq.gz
./ERR3130280_subreads.fastq.gz
./ERR3130281_subreads.fastq.gz
./ERR3130284_subreads.fastq.gz
./ERR3130285_subreads.fastq.gz
./ERR3130286_subreads.fastq.gz
./ERR3130287_subreads.fastq.gz
./ERR3130288_subreads.fastq.gz
./ERR3130289_subreads.fastq.gz
./ERR3130290_subreads.fastq.gz
./ERR3130291_subreads.fastq.gz
./ERR3130292_subreads.fastq.gz
