# Supplementary material for: The gene-rich genome of the scallop Pecten maximus
Source: Gigascience. 2020 Apr 30;9(5):giaa037. doi: 10.1093/gigascience/giaa037 (PMC7191990; doi:10.1093/gigascience/giaa037)
Supplement: giaa037_Supplemental_Files [file giaa037_supplemental_files.zip › SupplementaryFile2.zip!/AdditionalBlobplotFiles/multiplot_blobplot_phylum.Pecten_maximus.bestsum.phylum.p7.span.100.blobplot.multiplot.read_cov.bam0.pdf]

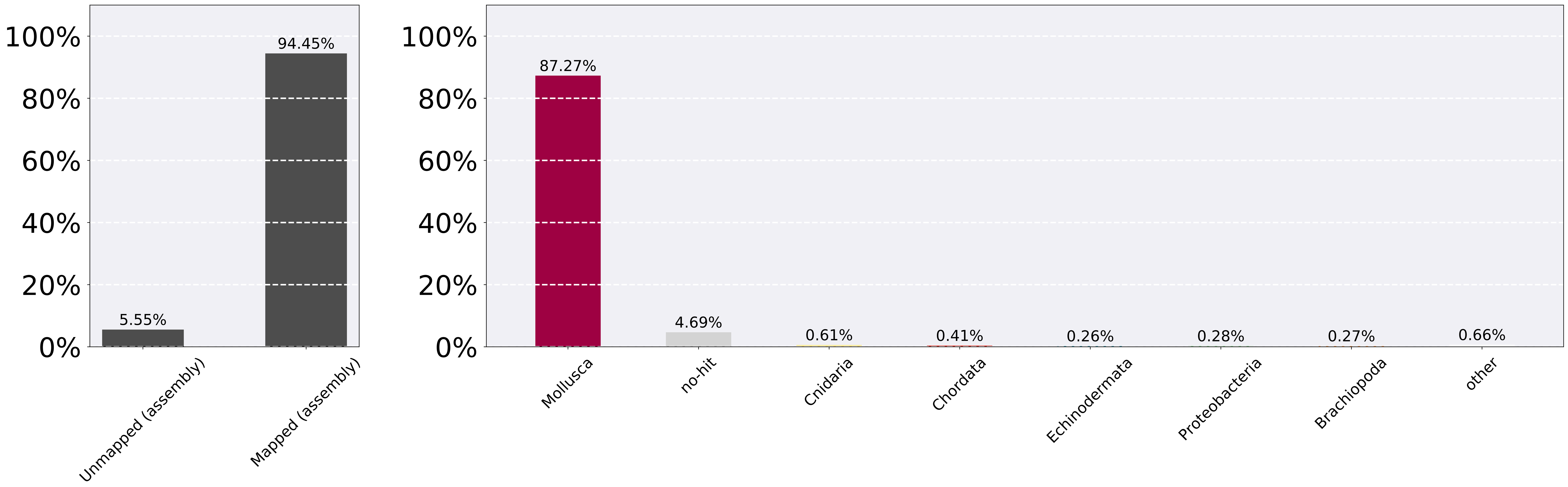

100%
80%
60%
40%
20%
0%
5.55%
94.45%
Unmapped (assembly)
Mapped (assembly)
87.27%
4.69%
0.61%
0.41%
0.26%
0.28%
0.27%
0.66%
Mollusca
no-hit
Cnidaria
Chordata
Echinodermata
Proteobacteria
Brachiopoda
other
