# Supplementary material for: The gene-rich genome of the scallop Pecten maximus
Source: Gigascience. 2020 Apr 30;9(5):giaa037. doi: 10.1093/gigascience/giaa037 (PMC7191990; doi:10.1093/gigascience/giaa037)
Supplement: giaa037_Supplemental_Files [file giaa037_supplemental_files.zip › SupplementaryFile2.zip!/AdditionalBlobplotFiles/standard_blobplot_phylum.Pecten_maximus.bestsum.phylum.p7.span.100.blobplot.bam0.pdf]

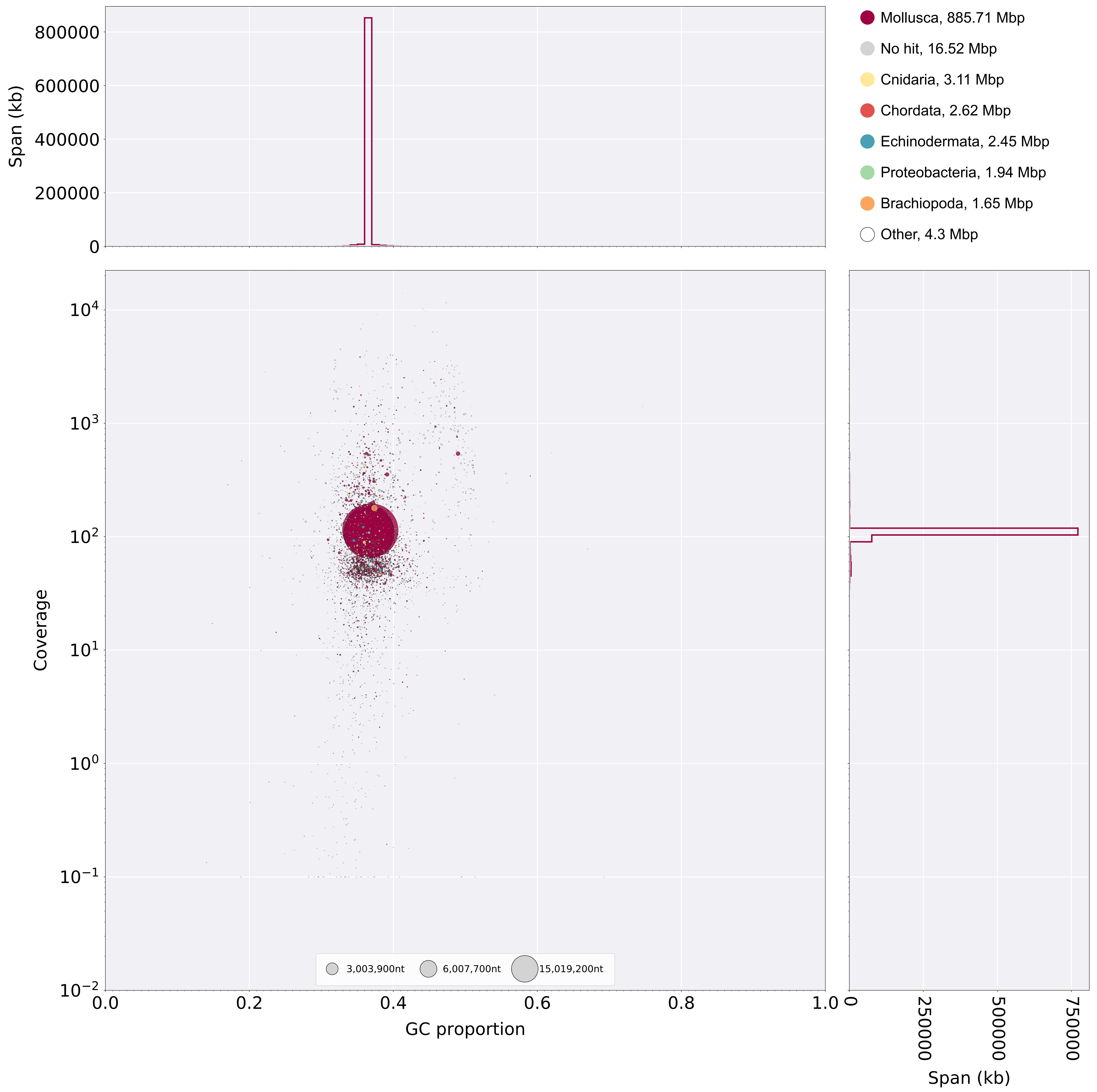

Mollusca, 885.71 Mbp
No hit, 16.52 Mbp
Cnidaria, 3.11 Mbp
Chordata, 2.62 Mbp
Echinodermata, 2.45 Mbp
Proteobacteria, 1.94 Mbp
Brachiopoda, 1.65 Mbp
Other, 4.3 Mbp
Span (kb)
Coverage
GC proportion
3,003,900nt
6,007,700nt
15,019,200nt
