# Supplementary material for: The gene-rich genome of the scallop Pecten maximus
Source: Gigascience. 2020 Apr 30;9(5):giaa037. doi: 10.1093/gigascience/giaa037 (PMC7191990; doi:10.1093/gigascience/giaa037)

standard_blobplot_superkingdom.Pecten_maximus.bestsum.superkingdom.p7.span.100.blobplot.bam0

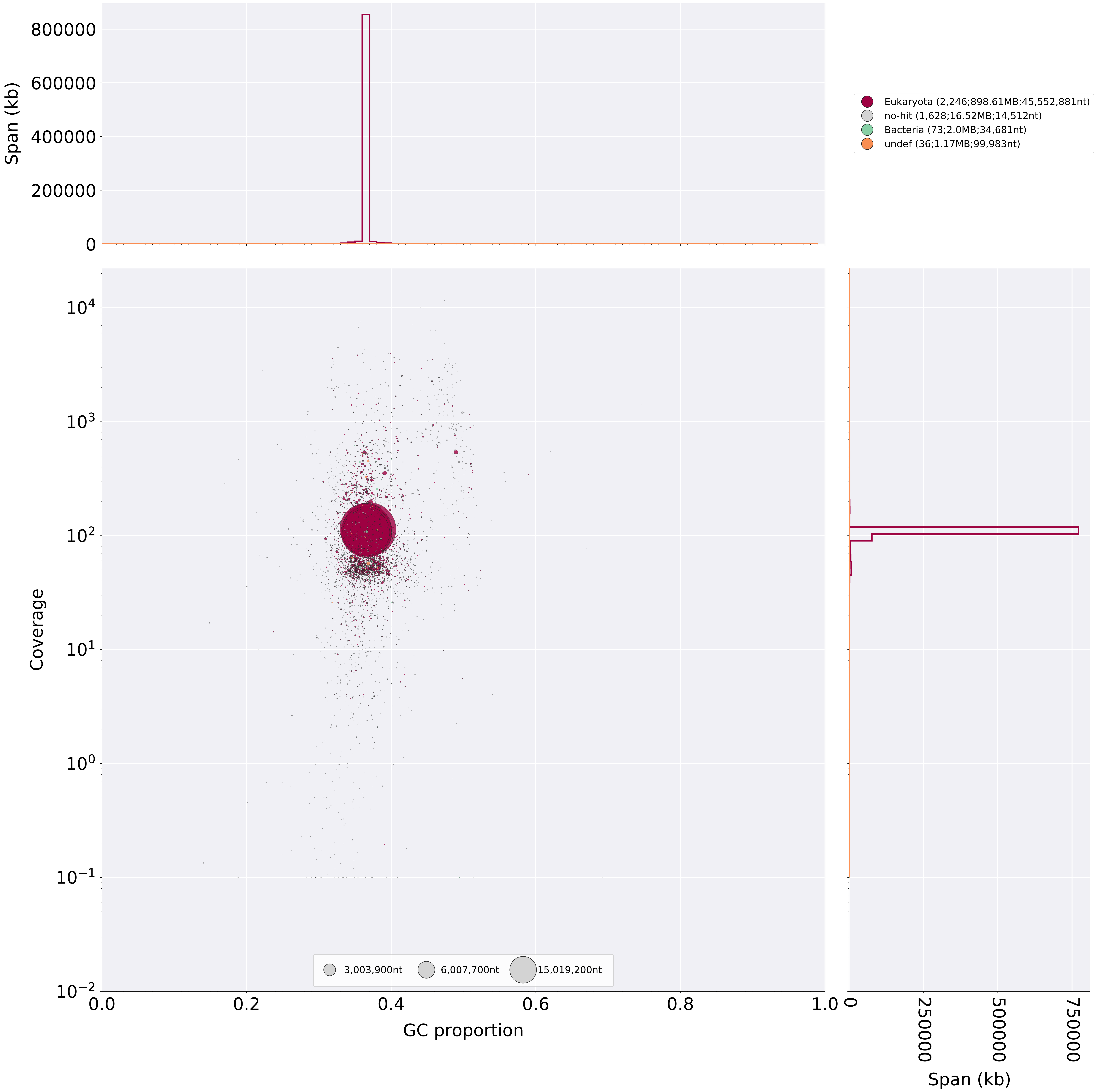

Supplement: giaa037_Supplemental_Files [file giaa037_supplemental_files.zip › SupplementaryFile2.zip!/AdditionalBlobplotFiles/standard_blobplot_superkingdom.Pecten_maximus.bestsum.superkingdom.p7.span.100.blobplot.bam0.pdf]
