# Supplementary material for: The gene-rich genome of the scallop Pecten maximus
Source: Gigascience. 2020 Apr 30;9(5):giaa037. doi: 10.1093/gigascience/giaa037 (PMC7191990; doi:10.1093/gigascience/giaa037)
Supplement: giaa037_Supplemental_Files [file giaa037_supplemental_files.zip › SupplementaryFile2.zip!/AdditionalBlobplotFiles/multiplot_blobplot_phylum.Pecten_maximus.bestsum.phylum.p7.span.100.blobplot.multiplot.bam0.1.Mollusca.pdf]

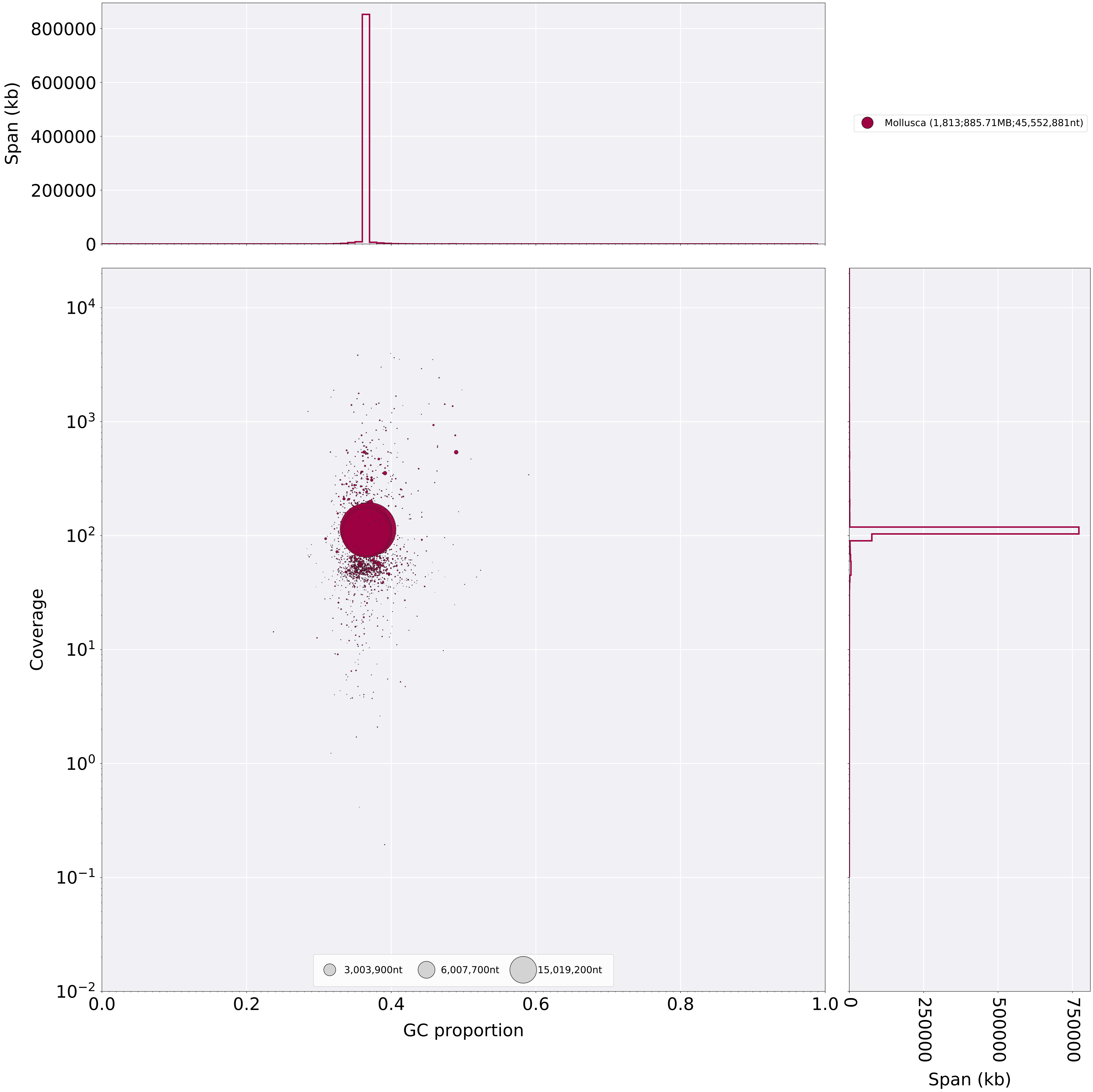

Span (kb)
800000
600000
400000
200000
0
Mollusca (1,813;885.71MB;45,552,881nt)
Coverage
$10^4$
$10^3$
$10^2$
$10^1$
$10^0$
$10^{-1}$
$10^{-2}$
3,003,900nt
6,007,700nt
15,019,200nt
0.0
0.2
0.4
0.6
0.8
1.0
GC proportion
0
250000
500000
750000
Span (kb)
