# Supplementary material for: The gene-rich genome of the scallop Pecten maximus
Source: Gigascience. 2020 Apr 30;9(5):giaa037. doi: 10.1093/gigascience/giaa037 (PMC7191990; doi:10.1093/gigascience/giaa037)
Supplement: giaa037_Supplemental_Files [file giaa037_supplemental_files.zip › SupplementaryFile2.zip!/AdditionalBlobplotFiles/multiplot_blobplot_phylum.Pecten_maximus.bestsum.phylum.p7.span.100.blobplot.multiplot.bam0.4.Chordata.pdf]

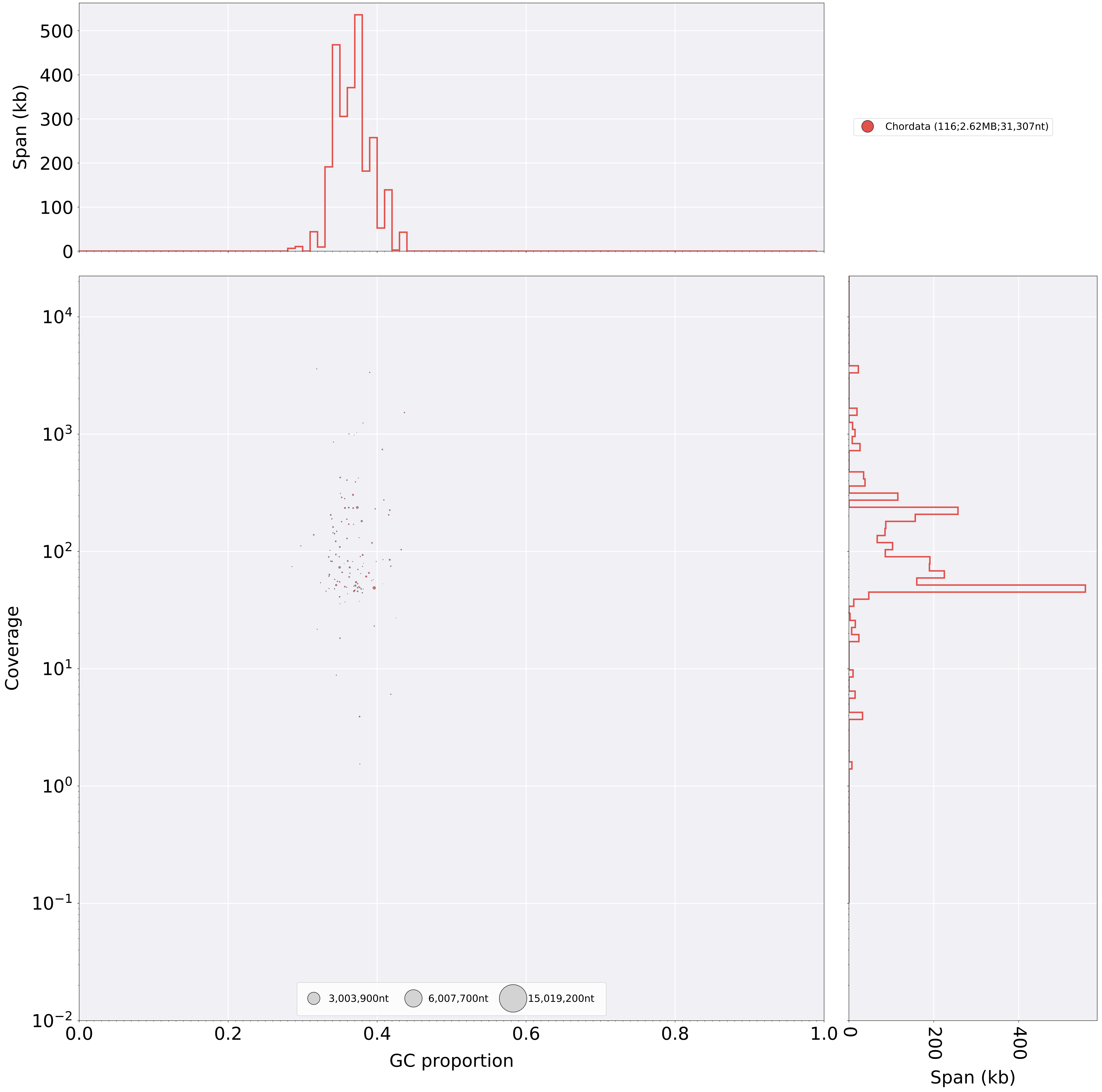

Span (kb)
500
400
300
200
100
0
Chordata (116;2.62MB;31,307nt)
Coverage
10^4
10^3
10^2
10^1
10^0
10^{-1}
10^{-2}
3,003,900nt
6,007,700nt
15,019,200nt
0.0
0.2
0.4
0.6
0.8
1.0
GC proportion
0
200
400
Span (kb)
